# Fecal Microbiota Was Reshaped in UCP1 Knock-In Pigs via the Adipose-Liver-Gut Axis and Contributed to Less Fat Deposition

**DOI:** 10.1128/spectrum.03540-22

**Published:** 2023-01-23

**Authors:** Jianfei Pan, Linya Chui, Tianxia Liu, Qiantao Zheng, Xuexue Liu, Lulu Liu, Ying Zhao, Lilan Zhang, Min Song, Jianlin Han, Jiaojiao Huang, Chaohua Tang, Cong Tao, Jianguo Zhao, Yanfang Wang

**Affiliations:** a State Key Laboratory of Animal Nutrition, Institute of Animal Science, Chinese Academy of Agricultural Sciences, Beijing, People’s Republic of China; b College of Animal Science and Technology, Qingdao Agricultural University, Qingdao, People’s Republic of China; c State Key Laboratory of Stem Cell and Reproductive Biology, Institute of Zoology, Chinese Academy of Sciences, Beijing, People’s Republic of China; d CAAS-ILRI Joint Laboratory on Livestock and Forage Genetic Resources, Institute of Animal Science, Chinese Academy of Agricultural Sciences (CAAS), Beijing, China; e Livestock Genetics Program, International Livestock Research Institute (ILRI), Nairobi, Kenya; Huazhong University of Science and Technology

**Keywords:** UCP1, fecal microbiota, HDCA, adipose tissue, pig

## Abstract

The relationship between the host gut microbiota and obesity has been well documented in humans and mice; however, few studies reported the association between the gut microbiota and fat deposition in pigs. In a previous study, we generated uncoupling protein 1 (UCP1) knock-in pigs (UCP1 pigs), which exhibited a lower fat deposition phenotype. Whether the gut microbiota was reshaped in these pigs and whether the reshaped gut microbiota contributes to the lower fat content remain unknown. Here, we revealed that the fecal microbiota composition and metabolites were significantly altered under both chow diet (CD) and high-fat/high-cholesterol (HFHC) diet conditions in UCP1 pigs compared to those in wild-type (WT) pigs. The abundance of *Oscillospira* and *Coprococcus* and the level of metabolite hyodeoxycholic acid (HDCA) from feces were observed to be significantly increased in UCP1 pigs. An association analysis revealed that *Oscillospira* and *Coprococcus* were significantly negatively related to backfat thickness. In addition, after fecal microbiota transplantation (FMT), the mice that were orally gavaged with feces from UCP1 pigs exhibited less fat deposition under both CD and high-fat diet (HFD) conditions, suggesting that the fecal microbes of UCP1 pigs participate in regulating host lipid metabolism. Consistently, HDCA-treated mice also exhibited reduced fat content. Mechanistically, we found that UCP1 expression in white adipose tissue alters the gut microbiota via the adipose-liver-gut axis in pigs. Our study provides new data concerning the cross talk between host genetic variations and the gut microbiota and paves the way for the potential application of microbes or their metabolites in the regulation of fat deposition in pigs.

**IMPORTANCE** This article investigated the effect of the ectopic expression of UCP1 on the regulation of fecal microbiota composition and metabolites and which alters the fat deposition phenotype. Bacteria, including *Oscillospira* and *Coprococcus*, and the metabolite HDCA were found to be significantly increased in feces of UCP1 pigs and had a negative relationship with backfat thickness. Mice with fecal microbiota transplantation phenocopied the UCP1 pigs under both CD and HFD conditions, suggesting that the fecal microbes of UCP1 pigs participate in regulating host lipid metabolism. Our study provides new data regarding the cross talk between host genetic variations and the gut microbiota and paves the way for the potential application of microbes or their metabolic production in the regulation of fat deposition in pigs.

## INTRODUCTION

The gut microbiota, also referred to as the hidden organ, harbors tens of trillions of microorganisms residing in the mammalian intestine that influence host physiology by regulating multiple processes, including nutrient absorption, inflammation, immune function, and anabolic balance (1, 2). Turnbaugh et al. performed one of the first studies definitively linking the gut microbiota to weight gain caused by an increase in the energy-harvesting capabilities of the “obese microbiota” (3). Numerous studies have established the association between gut microbiota and lean phenotype (or obesity) in humans and rodents. For example, Turnbaugh reported that “obese individuals” have an increased proportion of *Firmicutes* to *Bacteroidetes* than normal-weight individuals (4), while weight loss is associated with a reduced *Firmicutes*-to-*Bacteroidetes* ratio (5). Furthermore, germfree mice colonized with an microbiota isolated from genetically obese mice exhibited a greater percentage increase in body fat than those colonized with a “lean microbiota,” confirming that the gut microbiota regulates host fat storage as an environmental factor (6). The metabolic benefits observed in patients with obesity after fecal microbial transplantation (FMT) from lean donors indicate that gut microbiota management has become a new method of obesity treatment (7). All these results suggest the possibility of reducing fat content via the regulation of the gut microbiota.

Fat deposition is one of the most important economic traits in pigs, and breeding pigs with less fat deposition and more lean meat has always been the goal of pig breeding programs (8). Improvement in fatness traits can be achieved by many factors, including genetics, feed, and management (9). In addition to these well-known factors, the gut microbiota has been reported to contribute to host fat deposition in pigs (10–12). Some studies have attempted to identify the difference in gut microbiota composition between lean-type pig breeds and fat-type breeds; for example, Lu selected gut microbes for lean growth in a commercial crossbred population (Duroc × Large White × Landrace) from fecal samples by 16S rRNA gene sequencing and found that the alpha diversity of the fecal samples was negatively genetically correlated with backfat thickness (BFT) (10). Huang compared the fecal composition of wild boar, commercial crossbred pigs (lean-type breed), and Chinese domestic native pigs (fat-type breed) and found that crossbred pigs had a higher abundance of Streptococcus and *Lactobacillus* (13). Oh et al. reported that *Peptococcus* and *Eubacterium* were strongly positively correlated with body weight (BW) and average daily gain (ADG), whereas Treponema, *Desulfovibrio*, *Parabacteroides*, and *Ruminococcaceae*_unclassified were strongly negatively correlated with BW and ADG by comparing the fecal microbiota of high- and low-body-weight growing pigs aged 103 days (14). Chen et al. reported that Prevotella copri increases fat accumulation in Duroc pigs fed formula diets (11). Although these studies advanced our understanding of the role of gut microbes in fat deposition in pigs, the following problems remain: (i) most studies only revealed the correlation between fat phenotype and altered microorganisms, and no further functional validation has been performed; (ii) specific functional microorganism species that are related to lower fat deposition cannot be screened by the 16S rRNA gene method, and deep sequencing of bacteria is needed; and (iii) the screened microorganisms contribute to fat deposition, but not to reducing fat deposition, and cannot be used for the application of probiotics and feed additives for lower fat deposition (11). Therefore, pigs with homogeneous genetic backgrounds are needed to screen low-fat rate-associated gut microbiota and metabolites.

Uncoupling protein 1 (UCP1), a mitochondrial inner membrane carrier, plays an important role in brown adipose tissue (BAT)-mediated adaptive nonshivering thermogenesis (NST) (15, 16). The critical roles of UCP1 in thermoregulation and resistance to obesity have been widely studied (17, 18). It has been reported that UCP1 is functionally lost in pig lineages (19–21), providing a genetic explanation for the poor thermoregulation of piglets. In a previous study, we reconstituted the UCP1 gene in white adipose tissue (WAT) of Bama pigs (referred to as UCP1 pigs), which resulted in decreased fat deposition without affecting their physical activity and energy expenditure (22). Whether the gut microbiota was reshaped in UCP1 pigs and whether and to what extent the gut microbiota contributes to the lower fat content remain unknown. In addition, as a single gene-modified pig, UCP1 pigs represent an ideal animal material for screening the low-fat rate-associated gut microbiota and metabolites due to the homogeneous genetic background. Meanwhile, UCP1 pigs provide good material for studying the cross talk between host genetic variations and the gut microbiota.

In this study, we investigated the roles of UCP1 expression in WAT in reshaping the gut microbiota in Bama pigs by a 16S rRNA gene sequencing analysis and performed association studies between microorganism and phenotype data. FMT was conducted to test whether the phenotype of lower fat deposition in UCP1 pigs can be copied in mice. Significant different fecal metabolites between wild-type (WT) and UCP1 pigs were screened, and HDCA, a highly abundant metabolite in the feces of UCP1 pigs, was orally gavaged into mice to verify its function in host lipid metabolism. The potential mechanisms by which the ectopic expression of UCP1 alters the gut microbiota and affects host fat deposition were also investigated.

## RESULTS

### Fecal microbiota composition was altered in UCP1 pigs under CD conditions.

**FIG 1:**
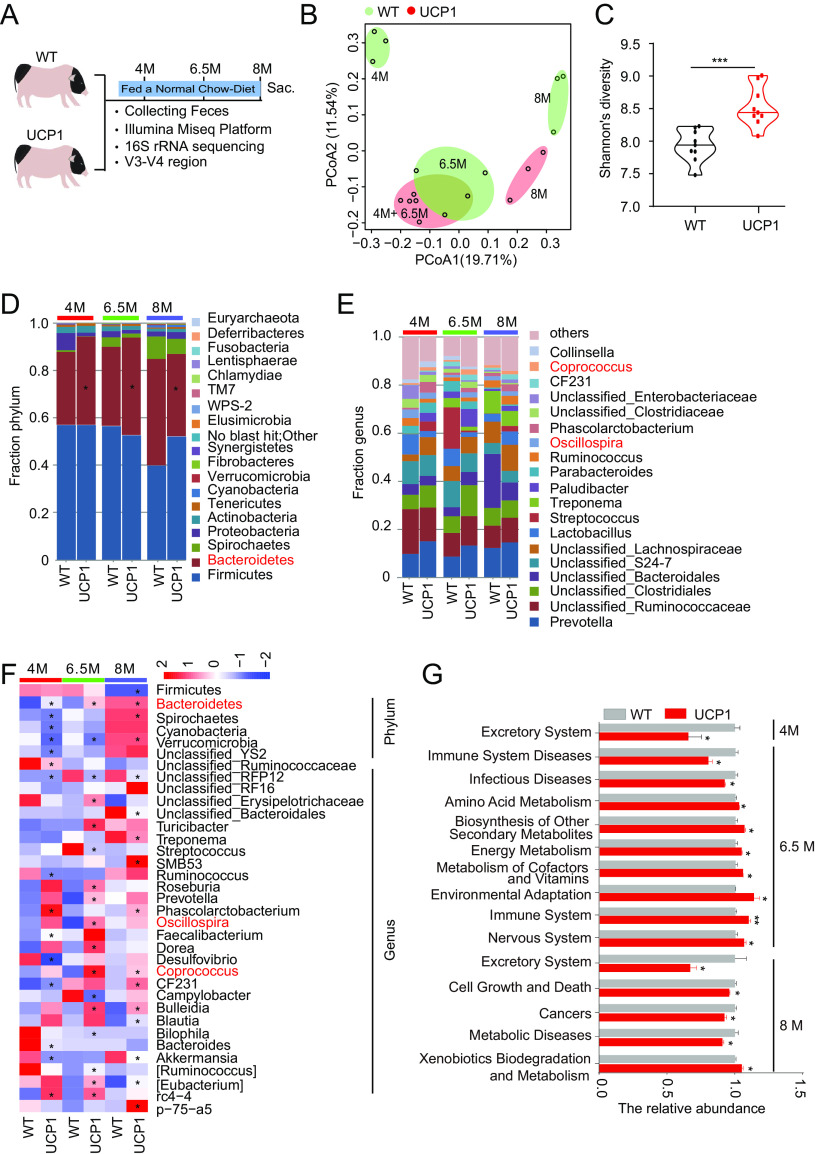
Ectopic expression of the UCP1 gene in the WAT of Bama pigs changes the fecal microbiota composition under chow diet conditions. (A) Schematic of the experimental design. (B) Unweighted UniFrac PCoA of the fecal microbiota from WT and UCP1 pigs at 4, 6.5, and 8 months of age. (C) Shannon’s diversity index of WT and UCP1 pigs. (D, E) Relative abundance of fecal microorganisms from WT and UCP1 pigs at the phylum level (D) and genus level (E) at each growth point. (F) Significantly different bacteria between UCP1 and WT pigs at the phylum and genus levels. (G) The predicted pathways differ between WT and UCP1 pigs at 4, 6.5, and 8 months of age. *, *P *< 0.05; **, *P *< 0.01; ***, *P *< 0.001.

It has been reported that host genetics shape mammalian gut microbiota (23), but whether and to what extent the gut microbiota are altered in UCP1 pigs are still unknown. Fecal samples were collected from WT and UCP1 pigs under chow diet (CD) conditions at the three indicated time points and subjected to a 16S rRNA gene sequencing analysis (Fig. 1A). Principal-coordinate analysis (PCoA) based on the unweighted UniFrac distance showed a clear separation between the samples from the WT and UCP1 pigs at each growth point (Fig. 1B), indicating significant alterations in the fecal microbiota composition in the UCP1 pigs. The alpha diversities differed between the WT and UCP1 pigs, and a significantly higher Shannon index was observed in the UCP1 pigs (*P* < 0.001) (Fig. 1C). Then, the bacterial relative abundance of all phyla and the top 20 genera were compared between the WT and UCP1 pigs at each growth point, and the results showed that the UCP1 pigs exhibited different bacterial composition patterns at each growth point. Specifically, a higher abundance of *Bacteroidetes* at the phylum level and increased levels of *Coprococcus* and *Oscillospira* at the genus level were observed in the UCP1 pigs at each growth point (Fig. 1D and E). To identify specific bacterial species that were characteristic of the WT and UCP1 pigs, we performed an analysis of the WT and UCP1 pigs at each growth point using the linear discriminant analysis (LDA) effect size (LEfSe) method. Compared to the WT pigs, the UCP1 pigs had significantly higher (*P *< 0.05) relative abundances of 9 taxa (led by *Phascolarctobacterium*) at 4 months of age, 24 taxa (led by *Oscillospira*) at 6.5 months of age, and 15 taxa (led by *Coprococcus*) at 6.5 months of age, while the WT pigs had significantly higher (*P *< 0.05) relative abundances of 27 taxa (led by *Akkermansia*) at 4 months of age, 14 taxa (led by Streptococcus) at 6.5 months of age, and 19 taxa (led by *Akkermansia*) at 6.5 months of age (see Fig. S1A in the supplemental material). The heatmap showed the relative abundance of 5 different bacteria at the phylum level and 30 different bacteria at the genus level across various time points between both pigs (Fig. 1F). Furthermore, we used PICRUSt to explore the potentially involved pathways in the UCP1 pigs, and the results showed that compared with the WT pigs, the microbiota in UCP1 pigs have a high abundance of functional annotation of amino acid metabolism, energy metabolism, immune system, and environmental adaptation at 6.5 months of age (Fig. 1G and Table S2). These results indicate that the ectopic expression of UCP1 in WAT changes the fecal microbiota composition in chow diet-fed pigs.

### Fecal microbiota composition was altered in UCP1 pigs under HFHC conditions.

**FIG 2:**
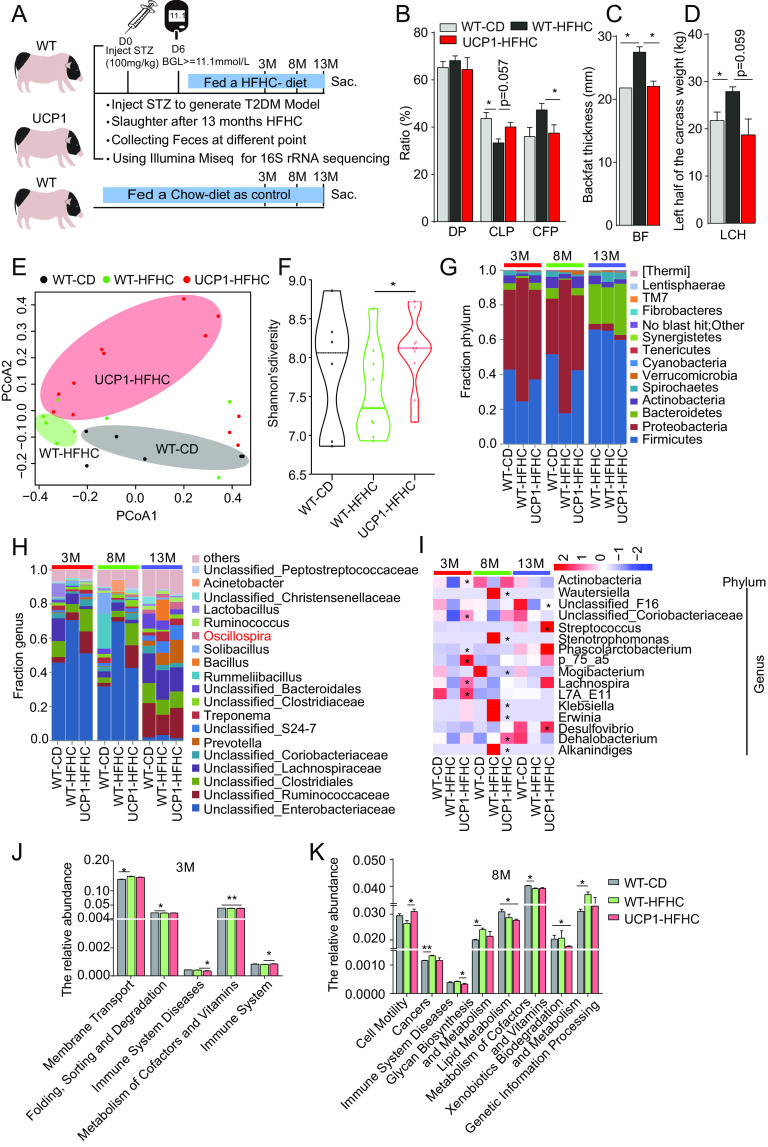
Altered fecal microbiota composition in UCP1 pigs fed an HFHC diet. (A) Schematic of the experimental design. (B) Histogram of the dressing percent (DP), lean meat in the left half of the carcass (CLP), and fat in the left half of the carcass (CFP) of three groups of pigs, including WT-CD, WT-HFHC, and UCP1-HFHC pigs. (C) Histogram of backfat thickness in the three groups of pigs. (D) Histogram of the left half of the carcass weight (LCH) of the three groups of pigs. (E, F) Unweighted UniFrac PCoA (E) and Shannon’s diversity index (F) of the fecal microbiota of the three groups of pigs at different treatment time points. (G, H) The relative abundance of fecal microbiota at the phylum level (G) and the genus level (H) in the three groups of pigs at different treatment time points. (I) Heatmap of significantly different bacteria among the three groups of pigs at different treatment time points. (J, K) The enrichment of predicted metabolic pathways among WT-CD, HFHC-fed WT, and UCP1 pigs after 3 months of the HFHC treatment (J) and 8 months of HFHC treatment (K). *, *P *< 0.05; **, *P *< 0.01.

In a previous study, UCP1 pigs were treated under high-fat/high-cholesterol (HFHC) diet conditions for 13 months to test the protective role of UCP1 on atherosclerosis (24). Furthermore, we measured the fat-related phenotypes, and the results showed that compared to HFHC-fed WT pigs (referred to as WT-HFHC pigs), the HFHC-fed UCP1 pigs (referred to as UCP1-HFHC pigs) exhibited a significantly lower carcass fat percentage (CFP) (3.45 ± 1.59% for UCP1-HFHC pigs versus 5.02 ± 0.75% for WT-HFHC pigs; *P *< 0.05) (Fig. 2B) and significantly thinner backfat thickness (BFT) (43.48 ± 7.77 mm for UCP1-HFHC pigs versus 52.03 ± 5.68 mm for WT-HFHC pigs; *P *< 0.05) (Fig. 2C). Meanwhile, histological analysis showed significantly smaller adipocytes in the UCP1-HFHC pigs than in the WT-HFHC pigs (Fig. S2A). Accordingly, the genes related to lipolysis, including *ATGL* and *HSL*, were upregulated in the UCP1-HFHC pigs (Fig. S2B). These data suggest that UCP1 pigs are resistant to HFHC-induced obesity.

Next, fecal samples were collected from WT pigs fed a CD diet and UCP1 and WT pigs fed an HFHC diet at various time points and subjected to 16S rRNA gene sequencing analysis (Fig. 2A). The PCoA analysis showed a clear separation among the groups at various time points (Fig. 2E), indicating significant alterations in the fecal microbiota composition in the UCP1 pigs fed the HFHC diet. The alpha diversities differed between the HFHC-fed WT and UCP1 pigs, and a significantly higher Shannon index was observed in the UCP1 pigs (*P* < 0.05) (Fig. 2F). As shown in Fig. 2G and H, different bacterial composition patterns were observed between the WT and UCP1 pigs after 3 months, 8 months, and 13 months of the HFHC diet treatment (Fig. 2G and H). In particular, similar fecal microbiota profiles were observed in the UCP1-HFHC pigs and WT-CD pigs after 3 months and 8 months of the HFHC treatment, while the WT-HFHC pigs exhibited a distinct bacterial composition pattern (Fig. 2G). This result was also observed at the genus level; for example, both the UCP1-HFHC pigs and WT-CD pigs had a lower level of unclassified_*Enterobacteriaceae* than the WT-HFHC pigs at various time points (Fig. 2H). To identify the specific bacterial species in each group of pigs, the LEfSe method was applied. The UCP1-HFHC pigs had significantly higher (*P *< 0.05) relative abundances of 9 taxa (led by *Phascolarctobacterium*) after 3 months of the HFHC diet, 2 taxa (L7A_E11 and *Dehalobacterium*) after 8 months of the HFHC diet, and 15 taxa (led by *Phascolarctobacterium*) after 13 months of the HFHC diet, while the WT-HFHC pigs had significantly higher (*P *< 0.05) relative abundances of 2 taxa (*Planococcaceae* and *Bacillales*) after 3 months of the HFHC diet and 13 taxa (led by *Flavobacteriales*) after 8 months of the HFHC diet; the WT-CD pigs had significantly higher (*P *< 0.05) relative abundances of 1 taxon (*Mogibacterium*) after 8 months of the HFHC diet (Fig. S2C to E). Next, we screened the bacteria with significant differences in abundance between the WT-HFHC and UCP1-HFHC pigs and built a heatmap across the three time points. In detail, at the phylum level, only *Actinobacteria* showed a significantly increased abundance in the UCP1-HFHC pigs compared to the WT-HFHC pigs after 3 months of the HFHC treatment. In addition, at the genus level, 16 different bacteria were decreased in the UCP1-HFHC pigs compared with those in the WT-HFHC pigs after exposure to the HFHC diet conditions (Fig. 2I). Similarly, we used PICRUSt to explore the potentially involved pathways in each group of pigs at various time points. The results showed that the microbiota in UCP1-HFHC group pigs have a low abundance of functional annotation of immune system diseases (primary immunodeficiency) and high abundance of functional annotation of the immune system (antigen processing and presentation) and cell motility (bacterial motility proteins) compared with the WT-HFHC group pigs after 3 months (*P *< 0.05) (Fig. 2J and Table S2) and 8 months (*P *< 0.05) (Fig. 2K and Table S2) of the HFHC diet treatment, respectively. These results indicate that the fecal microbiota composition was also changed in the UCP1 pigs under high-fat/high-cholesterol diet conditions.

### Oral administration of fecal microbes from UCP1 pigs prevents diet-induced obesity.

**FIG 3:**
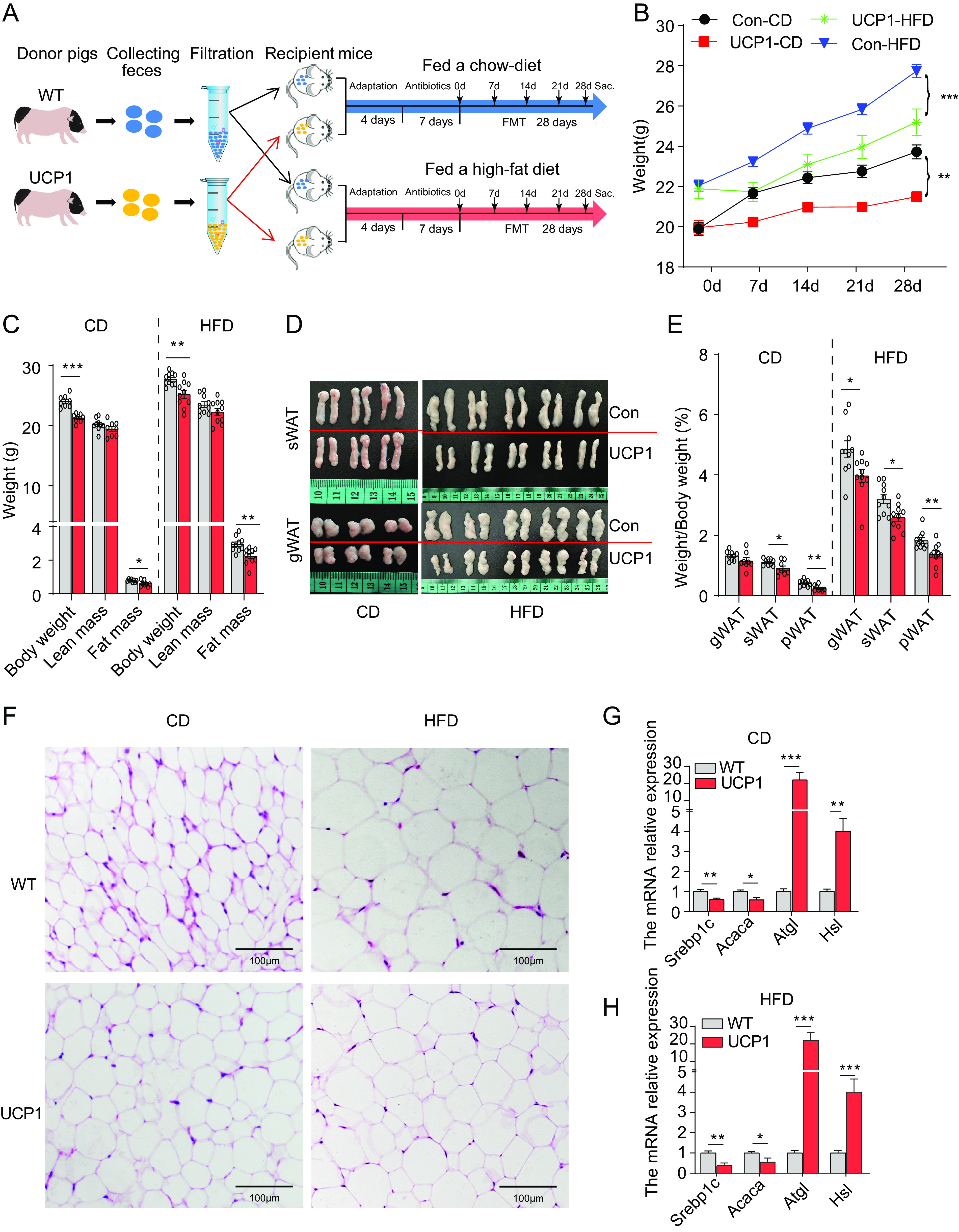
Oral administration of fecal microbes from UCP1 pigs prevents diet-induced obesity in mice. (A) Schematic design of FMT. (B) The body weights in the UCP1 group were significantly lower than those in the WT group under both CD and HFD conditions. (C) Body composition analyses of the UCP1 and WT groups after FMT for 28 days under both diet conditions. (D) Representative pictures of sWAT and gWAT from the UCP1 and WT groups after FMT for 28 days under both diet conditions. (E) Relative organ weights of the mice in the WT and UCP1 groups after FMT for 28 days under both diet conditions. (F) H&E images of the sWAT from the WT and UCP1 groups after FMT for 28 days under both diet conditions. (G, H) qPCR analysis of gene expression in sWAT from the WT and UCP1 groups after FMT for 28 days under CD conditions (G) and HFD conditions (H) (*n* = 5 mice/group), expressed as relative units using the threshold cycle (2^−ΔΔ^*^CT^*) method; *, *P *< 0.05; **, *P *< 0.01; ***, *P *< 0.001.

To explore whether the gut microbiota from UCP1 pigs contributes to their low-fat-rate phenotype, fecal microbiota transplantation was performed. Forty C57BL/6J male mice aged 4 months were randomly allocated to four groups, and all mice were treated with an antibiotic cocktail by oral administration every day for 7 days before FMT (Fig. 3A). Interestingly, a significantly lower body weight (BW) was observed in the mice treated with the fecal bacteria from UCP1 pigs (hereby referred to as the UCP1 group) than in the mice treated with the fecal bacteria from WT pigs (hereby referred to as the WT group) under both CD (Fig. 3B and C) and high-fat-diet (HFD) conditions (Fig. 3B and C). The body composition analyses revealed a markedly reduced fat mass in the UCP1 group under both CD and HFD conditions (Fig. 3C to E). The food intake and other peripheral organ weights in both groups were unchanged (Fig. S3A to C). In addition, the histological analysis of subcutaneous adipose tissue (sWAT) revealed that the mice in the UCP1 group contained smaller adipocytes under CD (Fig. 3F) and HFD conditions (Fig. 3F), indicating less lipogenesis or more lipolysis in the UCP1 group. Therefore, we examined the expression levels of genes involved in lipolysis and lipogenesis in sWAT from both groups of mice. Our data show that the lipolysis genes *Atgl* and *Hsl* were significantly upregulated in the UCP1 group, while lipogenesis-related genes, such as *Acaca* and *Srebp1c*, were significantly downregulated in sWAT from the UCP1 group under CD (Fig. 3G) and HFD (Fig. 3H) conditions. Altogether, these results indicate that fecal microbes or metabolites from UCP1 pigs participate in regulating host lipid metabolism.

### Abundances of *Oscillospira* and *Coprococcus* are negatively related to lower fat deposition phenotypes in UCP1 pigs.

**FIG 4:**
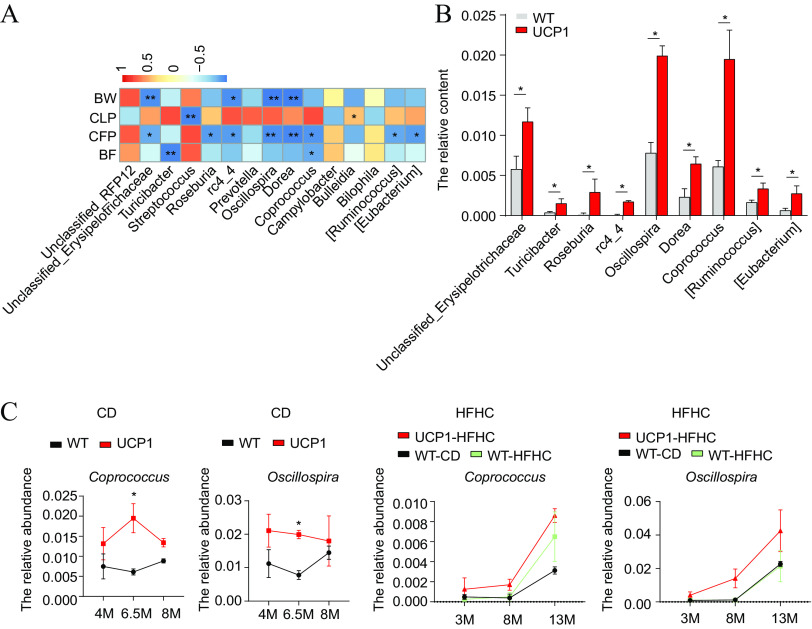
Abundances of *Oscillospira* and *Coprococcus* are negatively related to fat deposition phenotypes in UCP1 pigs. (A) Heatmap of Spearman’s correlations between the altered fecal microbes and phenotype data, such as BW, CLP, CFP, and BFT, of WT and UCP1 pigs at 6.5 months of age under CD. (B) Significantly induced fecal microbes in UCP1 pigs compared to those from WT pigs at 6.5 months of age under CD conditions. (C) Relative abundance of fecal *Oscillospira* and *Coprococcus* from WT and UCP1 pigs at each growth point under CD and HFHC diet conditions. *, *P *< 0.05; **, *P *< 0.01.

To further identify the fecal microbes that participate in regulating host lipid metabolism, Spearman’s correlation analyses were performed between the altered fecal microbes and the BW, carcass lean percentage (CLP), CFP, and BFT data collected from UCP1 pigs at 6.5 months of age under CD conditions. The results indicate that the abundances of unclassified_ *Erysipelontrichaceae*, cr4-4, *Oscillospira*, and *Dorea* were negatively correlated with CFP and BW (*P* < 0.05) (Fig. 4A). Streptococcus was negatively correlated with CLP (*P* < 0.01) (Fig. 4A), while *Bulleidia* was significantly positively correlated with CLP (*P* < 0.05) (Fig. 4A). *Turicibacter* and *Coprococcus* were significantly negatively correlated with BFT (*P* < 0.05) (Fig. 4A). *Roseburia*, *Coprococcus*, *Ruminococcus*, and *Eubacterium* were significantly negatively correlated with CFP (*P* < 0.05) (Fig. 4A). The microorganisms significantly and negatively related to BW, BFT, and CFP were considered potential low-fat-rate-associated candidates, and their relative contents are shown in Fig. 4B. Then, we examined their abundance across the different time points under both diet conditions and found that only *Oscillospira* and *Coprococcus* exhibited a higher abundance in the UCP1 pigs across different time points under both CD and HFHC diet conditions (Fig. 4C and Fig. S4). Thus, *Oscillospira* and *Coprococcus* are considered potential low-fat-rate phenotype-associated microorganisms.

### HDCA was screened as a lower fat deposition related metabolite in UCP1 pigs.

**FIG 5:**
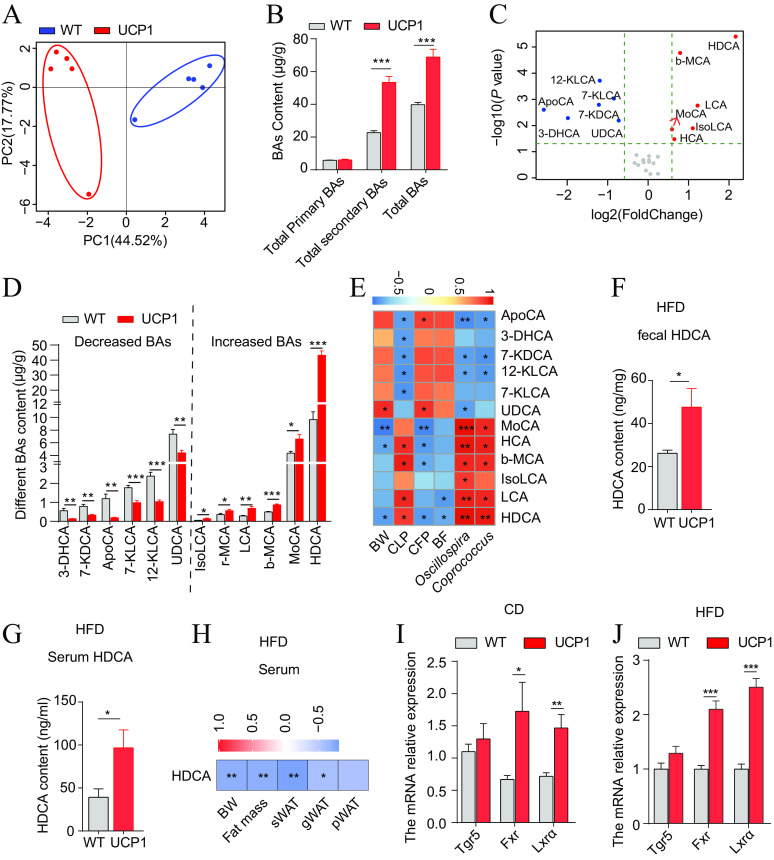
HDCA was screened as a low-fat deposition-related metabolite candidate in UCP1 pigs. (A) PCA plot of 26 BAs from fecal samples of 6.5-month-old UCP1 and WT pigs under CD conditions. (B) Total BAs, total primary BAs, and total secondary BAs between both groups of pigs. (C) Volcano plot of 26 BA profiles of fecal samples from UCP1 and WT pigs based on the criteria of a *P* value of <0.05 and fold change >1.5. (D) The different BAs between UCP1 and WT pigs. (E) Heatmap of Spearman’s correlations between the different fecal microbes and altered BAs. (F, G) HDCA content in feces (*n* = 6) (F) and serum (*n* = 8) (G) from WT and UCP1 groups of mice after FMT for 28 days under HFD conditions. (H) Heatmap of Spearman’s correlations between the HDCA content in serum and the BW, fat mass, weight of sWAT, gWAT, and perinephric white adipose tissue (pWAT) from WT and UCP1 groups of mice after FMT for 28 days under HFD conditions (*n* = 6 mice/group). (I, J) qPCR analysis of gene expression in sWAT from the WT and UCP1 groups after FMT for 28 days under CD conditions (I) and HFD conditions (J) (*n* = 5 mice/group) expressed as relative units using the 2^−ΔΔ^*^CT^* method; *, *P *< 0.05; **, *P *< 0.01; ***, *P *< 0.001.

It has been reported that bile acids (BAs), especially secondary bile acids derived from the gut microbiota, play important roles in the regulation of lipid, glucose, and energy metabolism (25). The enrichment of the energy metabolism pathway in UCP1 pigs suggests that bile acids may play a potential role in regulating fat metabolism (Fig. 1G). Therefore, an ultraperformance liquid chromatography–triple-quadrupole mass spectrometry (UPLC-TQMS)-based targeted metabolomics approach was used to analyze the bile acid profile of feces from WT and UCP1 pigs at 6.5 months of age. The PCA results revealed that the UCP1 pigs were distinct from the WT pigs, indicating a significant difference in the BA profiles between the groups (Fig. 5A). The total BAs and total secondary BAs were significantly increased in the UCP1 pigs, while the total primary BAs were unchanged (Fig. 5B). Twelve BAs were significantly altered in the feces from UCP1 pigs based on the criteria of a *P* value of <0.05 and fold change >1.5 in which HDCA, beta-muricholic acid (β-MCA), lithocholic acid (LCA), murocholic acid (MoCA), isolithocholic acid (IsoLCA) and hyocholic acid (HCA/γ-MCA) were significantly increased, and 12-ketolithocholic acid (12-KLCA), 7-ketolithocholic acid (7-KLCA), apocholic acid (ApoCA), 7-ketodeoxycholic acid (7-KDCA), 3-dehydrocholic acid (3-DHCA), and ursodeoxycholic acid (UDCA) were markedly decreased (Fig. 5C and D). The correlation analyses showed that HDCA, LCA, and IsoLCA were positively correlated with the abundance of *Oscillospira* (*P *< 0.01) and *Coprococcus* (*P *< 0.01) (Fig. 5E). Notably, HDCA was not only positively correlated with the abundance of *Oscillospira* and *Coprococcus* (*P *< 0.01) but also negatively correlated with BW, CFP, and BFT (*P *< 0.05). Furthermore, it is of interest to assess the HDCA level in FMT mice, and increased levels of HDCA were observed in both the feces (Fig. 5F) and serum (Fig. 5G) in the mice in the UCP1 group under HFD conditions. Consistent with the observation in pigs, the level of HDCA in serum was negatively correlated with BW (*P *< 0.01), fat mass (*P *< 0.01), sWAT weight (*P *< 0.01), and gonadal white adipose tissue (gWAT) weight (*P *< 0.05) (Fig. 5H), indicating that HDCA participates in regulating host fat deposition. Meanwhile, the expression of genes involved in bile acid signaling, including *Fxr* and *Lxrα*, was also increased in the sWAT of the UCP1 group of mice compared with the WT group of mice under CD (Fig. 5I) and HFD (Fig. 5J) conditions, indicating that HDCA may regulate host lipid metabolism by *Fxr*. Overall, HDCA could serve as a lower fat deposition related metabolite candidate in UCP1 pigs.

### HDCA administration reduces fat deposition.

**FIG 6:**
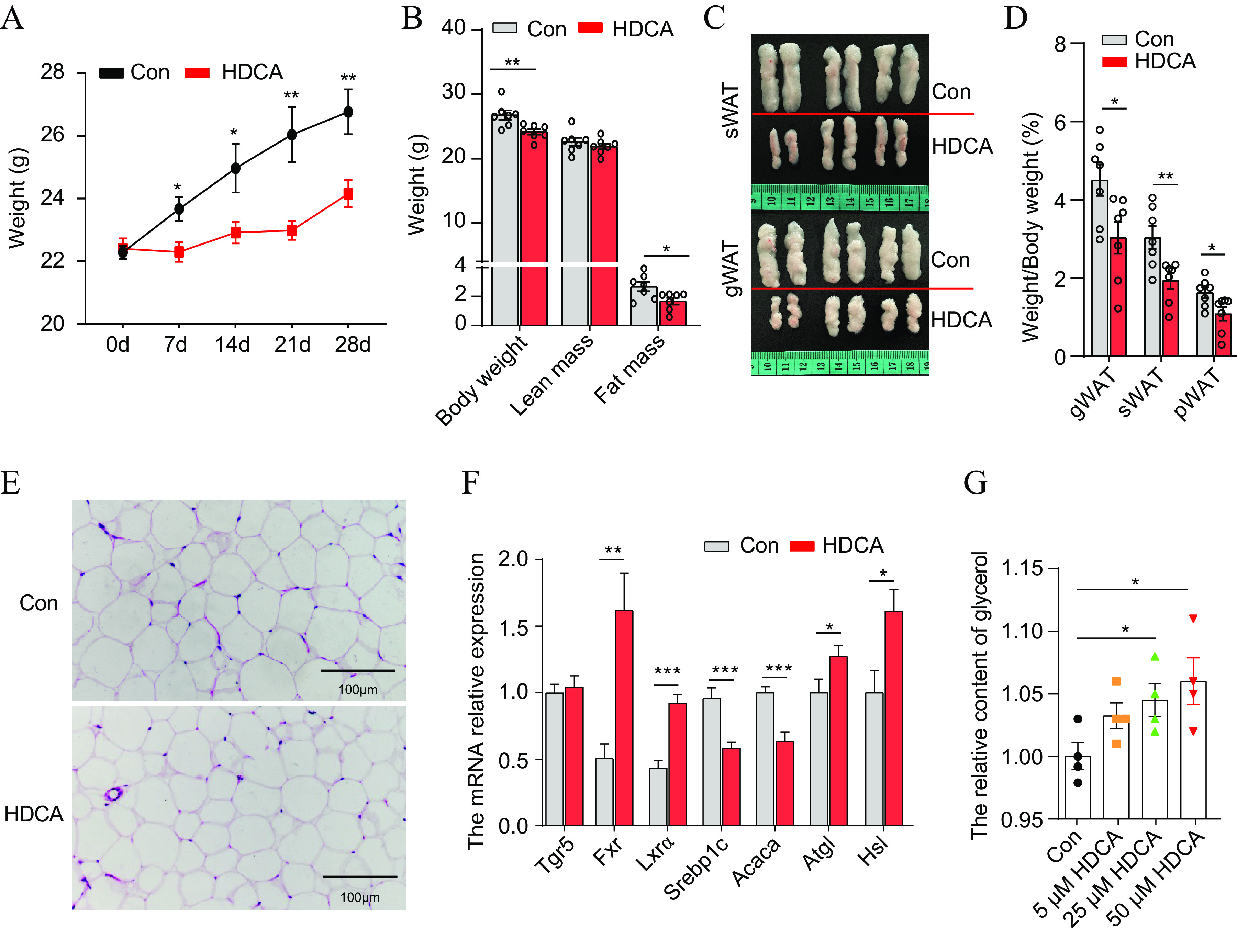
HDCA administration reduces fat deposition in mice. (A) The body weights of the mice in the HDCA group were decreased compared with those of the mice in the control (Con) group fed an HFD (*n* = 7). (B) Body composition analyses of the HDCA group and control group of mice (*n* = 7). (C) Representative pictures of sWAT and gWAT from the HDCA and WT groups. (D) Relative organ weights in the HDCA and control groups of mice (*n* = 7). (E) H&E staining of sWAT from the HDCA and control groups of mice. (F) qPCR analysis of gene expression in sWAT from the HDCA and WT groups of mice (*n* = 5 mice/group) expressed as relative units using the 2^−ΔΔ^*^CT^* method. (G) Glycerol content in the supernatant from 3T3-L1 cells after the HDCA treatment. Data are presented as the mean ± SEM. *, *P *< 0.05; **, *P *< 0.01; ***, *P *< 0.001.

To validate whether HDCA regulates host lipid metabolism, 20 4-week-old male mice were randomly divided into the following two groups (10 mice for each group): one group was treated with HDCA (100 μM/kg/day, dissolved in oleic acid) by oral administration (here referred to as the HDCA group), and the other group was treated with oleic acid (0.2 mL/20 g) as a control group (Fig. S5A). All mice were fed an HFD for 4 weeks (Fig. S5A). Interestingly, compared to the control group, the mice in the HDCA group exhibited a significantly lower body weight (Fig. 6A) and fat mass (Fig. 6B to D). The food intake (Fig. S5B) and other peripheral organ weights in both groups were unchanged (Fig. S5C). Furthermore, the histological analysis revealed smaller adipocytes in sWAT from the mice in the HDCA group (Fig. 6E), indicating decreased lipogenesis or enhanced lipolysis of adipose tissue. Therefore, we examined the expression of genes involved in lipolysis and lipogenesis. Our data show that the lipolysis genes *Atgl* and *Hsl* were significantly upregulated (Fig. 6F), while the lipogenesis-related genes *Acaca* and *Srebp1c* were significantly downregulated in the sWAT of the HDCA group of mice compared with those in the control group of mice (Fig. 6F). Meanwhile, we also observed that the expression levels of genes involved in bile acid signaling, including *Fxr* and *Lxrα*, were upregulated in the sWAT of the HDCA group compared with those in the WT group (Fig. 6F). Altogether, these data indicate that HDCA participates in host lipid metabolism with increased lipolysis and decreased lipogenesis through bile acid signaling. In addition, we estimated the effect of HDCA on lipogenesis and lipolysis in 3T3-L1 cells and found that HDCA treatment did not affect the differentiation efficiency and lipogenesis of 3T3-L1 cells (Fig. S5D and E) but increased lipolysis, as evidenced by significantly increased glycerol levels (Fig. 6G; Fig. S5F). Altogether, HDCA administration reduces fat deposition in mice by increasing lipolysis in sWAT.

### UCP1 expression in WAT shapes the gut microbiota via the adipose-liver-gut axis in pigs.

**FIG 7:**
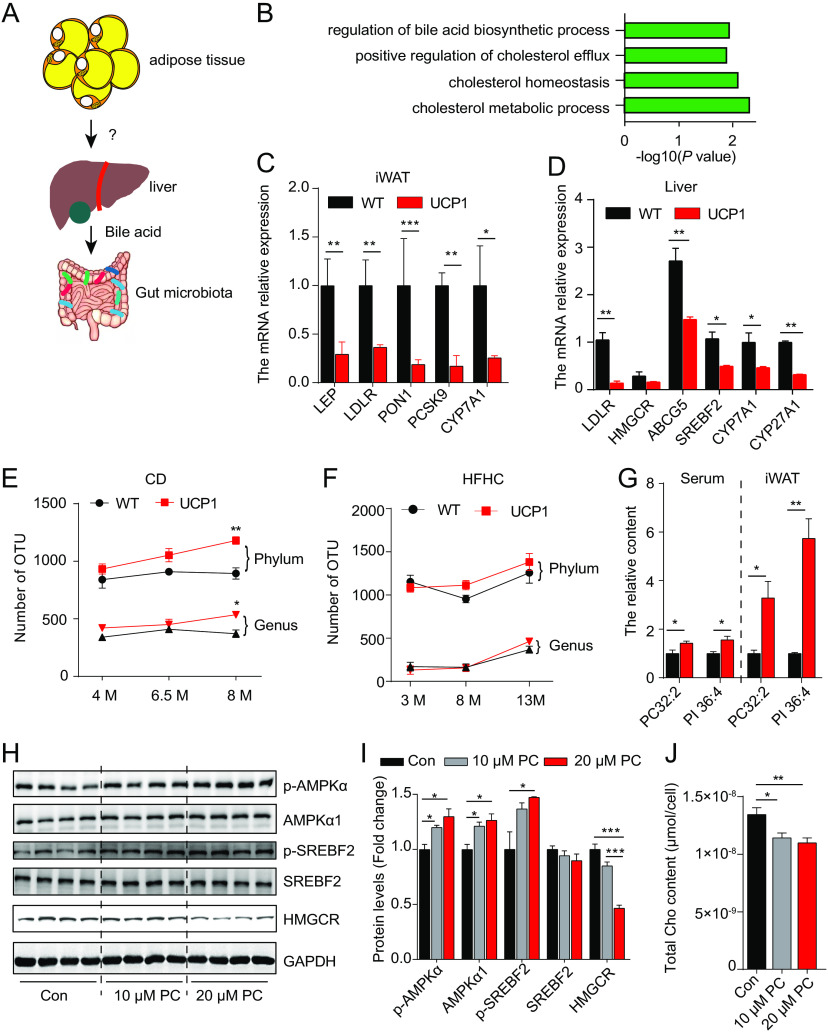
UCP1 expression in WAT shapes the gut microbiota via the adipose-liver-gut axis in pigs. (A) Model hypothesizing that UCP1 expression in WAT shapes the gut microbiota by the adipose-liver-gut axis in pigs. (B) Gene ontology (GO) terms of downregulated genes enriched in cholesterol homeostasis and bile acid biosynthetic processes in UCP1 pigs. (C) Decreased expression levels of cholesterol homeostasis and bile acid biosynthetic processes in fat tissues from UCP1 pigs. (D) mRNA relative expression of key genes related to cholesterol homeostasis and bile acid biosynthetic processes in the livers of WT and UCP1 pigs fed an HFHC diet. (E, F) Numbers of OTUs at the phylum and genus levels in UCP1 pigs under CD conditions (E) and HFHC conditions (F). (G) PC32:2 and PI36:4 contents in iWAT and serum from UCP1 and WT pigs. (H) Western blot results of key genes related to cholesterol homeostasis after treatment with 10 μM or 20 μM PC in HepG2 cells. (I) Protein level analysis of panel H. (J) Cholesterol content in HepG2 cells after 10 μM or 20 μM PC treatment for 24 h. Data are presented as the mean ± SEM. *, *P *< 0.05; **, *P *< 0.01; ***, *P *< 0.001.

It has been reported that the bile acid-gut microbiota axis contributes to obesity susceptibility (26). The altered fecal microbiota (Fig. 1B) and bile acid profile (Fig. 5A) in the feces from UCP1 pigs suggest the possibility of the existence of a liver-gut axis in UCP1 pigs. Therefore, we hypothesized that the expression of UCP1 in WAT shapes the gut microbiota via the adipose-liver-gut axis (Fig. 7A). To investigate how UCP1 expression in WAT shapes the host gut microbiota, we assessed the genome-wide transcriptional data that we previously obtained from inguinal subcutaneous adipose tissue (iWAT) of cold-treated WT and UCP1 pigs (27). Interestingly, the genes involved in cholesterol homeostasis (*LDLR*, *PCSK9*, *MED13*, *PON1*, *ABCG1*, *LEP*, and *CEBPA*) and regulation of the bile acid biosynthetic process (*CYP7A1* and *SIRT1*) were significantly downregulated in iWAT from UCP1 pigs compared to those from WT pigs (Fig. 7B and C). It has been reported that cholesterol is mainly synthesized in the liver and primarily converted into bile acids by several enzymatic reactions (28). Then, by quantitative real-time PCR (qPCR), we examined the expression of key genes involved in cholesterol and bile acid synthesis in the livers of WT and UCP1 pigs under HFHC conditions. The results showed that cholesterol homeostasis-related genes, including *LDLR*, *ABCG5*, and *SREBF2*, and the rate-limiting enzymes of bile acid synthesis (*CYP7A1* and *CYP27A1*) were significantly downregulated in the liver of UCP1 pigs (Fig. 7D), but there were no changes in key genes responsible for the uptake of cholesterol, *NPC1L1*, in the gut (Fig. S6A). Studies have reported that bile acid is a significant host factor shaping the gut microbiota in mice and rats (29, 30). The decreased cholesterol level in the liver implies a low level of primary BAs in the duodenum and an increased level of gut microbes in the small intestine due to the attenuated ability of BAs to control gut bacterial overgrowth (31). Therefore, the number of gut microbiota constituents in WT and UCP1 pigs was calculated by 16S rRNA gene sequencing, and the results showed more operational taxonomic units (OTUs) at the phylum and genus levels in the UCP1 pigs than in the WT pigs under both CD (Fig. 7E) and HFHC (Fig. 7F) conditions. Our previous data show that UCP1 expression in WAT changed the lipid profiles of iWAT (27). We hypothesize that the altered lipids in WAT are secreted into the circulation to regulate cholesterol biosynthetic processes in the liver. Two lipid species, PC32:2 and PI36:4, were found to be significantly increased in the iWAT and circulation of UCP1 pigs based on serum and adipose lipidomics, with the criteria of a *P* value of <0.05 and fold change of >1.5 (Fig. 7G; Fig. S6B). Then, we treated HepG2 cells with phosphatidylcholine (PC) and phosphatidylinositol (PI) to determine whether cholesterol synthesis-related genes and the cholesterol levels were changed. We found that PC could decrease the expression levels of the key cholesterol synthesis gene *HMGCR* (Fig. 7H) and its regulatory sterol regulatory element binding protein 2 (SREBP2, also named SREBF2) (Fig. 6H). A previous study reported that AMPK directly phosphorylates SREBP2, which inhibits the nuclear translocation of SREBP2 and downregulates HMGCR transcription (32). Thus, we examined the expression of genes that regulate HMGCR expression, and the results showed that the protein levels of p-AMPKa (Thr172), AMPKa1, and p-SREBF2 (Ser455) were increased after the PC treatment (Fig. 7H to I). Decreased cholesterol levels (Fig. 7J) were also observed in the PC-treated HepG2 cells but not the PI-treated HepG2 cells (Fig. S6C). The data described above indicate that PC treatment may downregulate cholesterol levels by AMPK signaling. These results show that the exotic expression of the UCP1 gene in WAT shapes the gut microbiota via the adipose-liver-gut axis in pigs.

### HDCA content in feces negatively correlates with BFT in pigs.

**FIG 8:**
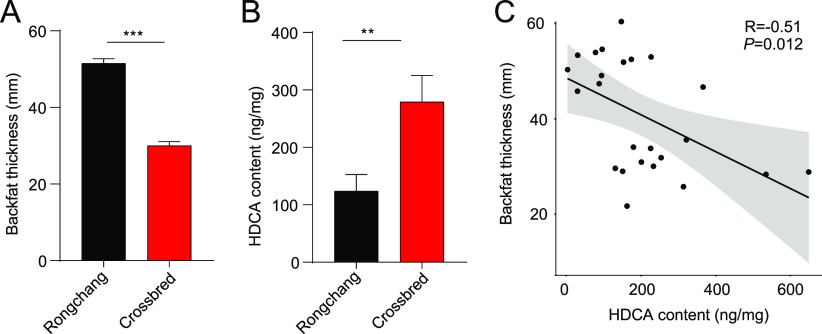
HDCA content in feces negatively correlates with BFT in pigs. (A) Average BFT of Rongchang and crossbred pigs. (B) Fecal HDCA content in Rongchang and crossbred pigs fed formula diets (*n* = 15 for each group). (C) The linear regression assays showed that the fecal HDCA content negatively correlates with BFT. The correlation coefficient (*R*) and *P* value by nonparametric Spearman’s rank correlation are shown. The gray area represents the fitting curve. Data are presented as the mean ± SEM. **, *P *< 0.01; ***, *P *< 0.001.

The data described above indicate that the oral administration of HDCA reduces host fat deposition in mice. Whether the fecal HDCA levels negatively correlate with fat deposition in pigs is unclear. We found that commercial crossbred pigs, a lean-type pig breed, had significantly thinner backfat than Rongchang pigs, a Chinese native fat-type pig breed (*P* < 0.001) (Fig. 8A). A significantly higher content of fecal HDCA was observed in the crossbred pigs (*P* < 0.01) (Fig. 8B). Most remarkably, a significant negative correlation was observed between the fecal HDCA content and BFT (Spearman analysis; *R *= −0.51, *P *= 0.012) (Fig. 8C). Thus, our data suggest that the fecal HDCA content might be a potential indicator of fat deposition in pigs.

## DISCUSSION

The host gut microbiota is considered a complex ecosystem composed of distinct microbial populations. Accumulating evidence has shown that host genetics reshape the gut microbiota (33, 34). For example, the genetic deletion of myeloid differentiation factor 88 in T cells leads to the loss of *Ruminococcus* spp. (35). Intestine-specific HIF-2α ablation resulted in less Bacteroides vulgatus, greater Ruminococcus torques, and elevated taurine-conjugated cholic acid (TCA) and deoxycholic acid (DCA) levels, which activated the expression of UCP1 and WAT-mediated thermogenesis (36). These observations suggest that the microbiota composition of UCP1 knock-in pigs may be altered. Our study shows that *Firmicutes*, *Bacteroidetes*, *Spirochaetes*, and *Proteobacteria* at the phylum level are the four most abundant bacteria in both WT and UCP1 pigs under both CD and HFHC conditions, which is consistent with other fecal microbiota studies in pigs (14, 37). A higher abundance of *Bacteroidetes* was observed in 4- and 6.5-month-old UCP1 pigs, which is consistent with the results of low-fat diet-fed Ossabaw pigs (38), indicating that UCP1 pigs have the same pattern of fecal gut microbiota as lean-type pigs. Consistently, after the HFHC diet treatment, similar microbiota profiles were observed between the UCP1-HFHC pigs and WT-CD pigs. The low abundance of microbiota with functional annotation of immune system diseases (primary immunodeficiency) and infectious diseases (Staphylococcus aureus infection) and high abundance of microbiota with functional annotation of the immune system (antigen processing and presentation) were observed in UCP1 pigs, which suggested that UCP1 pigs seem to have a healthier gut, although more immune-related work needs to be performed.

The development of gut microbiome technology has made it possible to screen the low-fat-rate-associated gut microbiota and metabolites. In our study, a significant negative correlation was observed between *Coprococcus* and *Oscillospira* and BFT in UCP1 pigs (Fig. 5E), suggesting that both microbes were low-fat-rate phenotype-related genera. *Coprococcus* is a butyrate-producing bacterium that has been reported to be associated with higher quality-of-life indicators (39). Although the involvement of *Coprococcus* in energy and fat metabolism has rarely been reported, a decreased abundance of *Coprococcus* has been observed in patients with Parkinson’s disease (40) and autism spectrum disorder (41). A positive correlation was observed between *Coprococcus* and HDCA and LCA in our data (Fig. 5E). Consistently, Lin reported that the *Coprococcus* levels were positively correlated with fecal DCA in a rat obesity model (42), indicating that *Coprococcus* may participate in BA metabolism.

*Oscillospira* is an enigmatic bacterial genus that has never been cultured. Evidence has shown that *Oscillospira* is positively associated with leanness and health, could be a predictor of a low body mass index (BMI) (43, 44), and could be considered a candidate for next-generation probiotics (45). Although multiple species and strains of *Oscillospira* in the human gut have been isolated (46), no specific species have been identified in pigs (47). A positive correlation between *Oscillospira* and HDCA and LCA was observed in our study (Fig. 5E), which is consistent with the data of gallstone patients scheduled for cholecystectomy before and after in which *Oscillospira* was positively correlated with secondary BAs (48). All of these observations indicate that *Oscillospira* could be regarded as a potential low-fat candidate microorganism. Due to the limitation of 16S rRNA gene sequencing, no specific related species of either microorganism were identified; currently, a metagenome analysis at the species level and loss- or gain-of-function studies of bacteria are required for further study and application in reducing pig fat deposition in the future.

Bile acids are the products of cholesterol (primary bile acids) and bacteria (secondary bile acids), whose contents are changed in many diseases and participate in regulating hepatic lipid, glucose, and energy homeostasis by nuclear receptors and G protein-coupled receptor (GPCR) signaling (49). In our study, bile acid pools were changed with significantly increased HDCA, LCA, and iso-LCA in UCP1 pigs (Fig. 5C and D). It has been reported that Iso-LCA is produced by Eubacterium lentum and Clostridium perfringens and can be found in human serum and urine (50). Consistent with this report, an increased *Eubacterium* and *Clostridium* abundance at the genus level was also observed in the feces of the UCP1 pigs under CD conditions. HDCA is a secondary bile acid, and it has been reported that serum HDCA levels are significantly negatively correlated with BMI (51). Consistently, in our study, HDCA was found to be significantly negatively correlated with BW, CFP, and BFT and significantly positively correlated with *Oscillospira* and *Coprococcus.* In fact, the effect of HDCA supplementation on the growth of animals has been studied. Powdered HDCA was mixed in a high-caloric diet and given to mice for 4 weeks, and the results showed that the HDCA treatment had no effect on the body weight of the mice (52). Similar work has been performed in piglets, and the average daily gain and average daily feed intake of the piglets were not affected (53). However, in our study, the mice orally treated with HDCA exhibited a significantly lower body weight (Fig. 6A) and decreased fat mass (Fig. 6B to D). The contradictory results may be due to the different diet types, different methods of HDCA administration, and different treatment times and doses.

HDCA was screened as a low fat deposition related metabolite candidate in UCP1 pigs, and it is of interest to further investigate the relationship between HDCA and backfat thickness in other pig populations. Crossbred pigs (lean-type breed) and Rongchang pigs, a famous Chinese native fat-type breed, were used for the validation of the observation in UCP1 pigs. Excitingly, we found that the fecal HDCA contents are significantly related to backfat thickness. However, more critical experiments are required to determine whether HDCA can be used as a feed additive to reduce fat deposition in pigs. For example, the association studies need to be validated in large populations, and HDCA needs to be used to feed pigs to determine its role *in vivo*.

In summary, this study revealed that the expression of UCP1 in the WAT of pigs changed the gut microbiota composition via the adipose-liver-gut axis and altered the fecal microbiome. Metabolites reduced the fat contents by promoting lipolysis of WAT. The bacteria *Oscillospira* and *Coprococcus* were screened as potential low-fat-rate-phenotype genera, and HDCA has the potential to decrease fat deposition in pigs. Although further work is needed to investigate the function of these bacteria and HDCA, our findings pave the way for the further application of these bacteria and metabolites as feed additives for lower fat deposition in the future.

## MATERIALS AND METHODS

### Ethics statement and animal housing.

All experiments involving pigs and mice were performed according to the guidelines for the care and use of laboratory animals established by the Beijing Association for Laboratory Animal Science and were approved by the Animal Ethics Committee of the Institute of Animal Science (IAS2021-33), Chinese Academy of Agricultural Sciences (CAAS). The detailed information on the UCP1 pigs and tissues used in this study was previously described (22).

### HFHC diet treatment and carcass trait measurements.

Detailed information on the HFHC treatment has been previously described (24). In brief, a cohort of 8-month-old male UCP1 pigs and wild-type pigs was fed a high-fat and high-cholesterol diet (HFHC; basic diet with 15% lard and 1% bile salts) for 13 months, and a cohort of male WT pigs was fed a chow diet as a control group (Fig. 2A). All pigs were slaughtered, and the carcass traits were measured and sampled after 13 months of HFHC.

The pigs under the HFHC conditions were fasted for 24 h before slaughter and anesthetized to death. At slaughter, the carcass was split longitudinally, and the head, hair, feet, and viscera (except for the leaf fat and kidney) were removed. The carcass weight was recorded. The dressing percentage of an individual animal was defined as the carcass weight divided by the live weight. The measurements of BFT at the midline were conducted with a Vernier caliper at the scapular margin, last rib, and lumbosacral junction, and the average BFT of the three points was used. The left half of the carcass was dissected to obtain lean meat, fat, skin, and bone. The percentage of each part was calculated by dividing each by the weight of the left half of the carcass.

### DNA extraction and microbial community sequencing.

Fecal samples were collected directly into sterile tubes, and DNA extraction and purification were performed using a Tiangen DNA stool minikit (Tiangen; catalog no. DP328) and a QIAquick gel extraction kit (Qiagen; catalog no. 28706), respectively. Illumina-compatible PCR amplification of the variable 3 and 4 (V3-V4) region of the 16S rRNA gene was completed in each sample. The Illumina MiSeq platform was used to sequence the DNA products of this PCR amplification (MiSeq reagent kit V3, 600 cycles). More detailed information on the cDNA library construction, sequencing of paired-end (PE) libraries, and quality control was presented in a previous study (54). OTUs were grouped using abundant OTUs based on 97% similarity. The 2013 version of the Greengenes reference database (release gg_13_8_99; http://greengenes.secondgenome.com/) was used to assign taxonomy to the OTUs Ribosomal Database Project (RDP; release 11.1; http://rdp.cme.msu.edu/) classifier in the Quantitative Insights Into Microbial Ecology version 2 (QIIME2). QIIME2 and R scripts were used to calculate the beta diversity using Bray-Curtis dissimilarity and a principal-coordinate analysis to generate plots of the taxonomy data and perform statistical tests. The microbial function was predicted using PICRUSt. A linear discriminant analysis (LDA) of effect size (LEfSe) was conducted to define the biomarkers of each group at the website http://huttenhower.sph.harvard.edu/galaxy/. Some of the R scripts are publicly available at https://github.com/pjf124/MicroSpectrum.

### FMT.

Specific-pathogen-free (SPF) 4-week-old male C57BL/6J mice were purchased from Beijing SBF Bio-Technology Co., Ltd. (Beijing, China) and maintained under a standard 12-h dark cycle with free access to water and diet. Forty C57BL/6J male mice were randomly divided into 4 groups for FMT, namely, (i) the WT-CD group, which was orally administered fecal bacteria derived from WT pigs under CD conditions; (ii) the UCP1-CD group, which was orally administered fecal bacteria derived from UCP1 pigs under CD conditions; (iii) the WT-HFD group, which was orally administered fecal bacteria derived from WT pigs under HFD conditions; and (iv) the UCP1-HFD group, which was orally administered fecal bacteria derived from UCP1 pigs under HFD conditions. All mice were treated with combined antibiotics containing 100 μg/mL neomycin (Solarbio; catalog no. N8090), 50 μg/mL streptomycin (Solarbio; catalog no. S8290), 100 U/mL penicillin (Solarbio; catalog no. P8420), 50 μg/mL vancomycin (Solarbio; catalog no. V8050), 100 μg/mL metronidazole (Solarbio; catalog no. M8060), 1 mg/mL bacitracin (Solarbio; catalog no. B8181), 125 μg/mL ciprofloxacin (Solarbio; catalog no. C9710), 170 μg/mL gentamicin (Solarbio; catalog no. G8170), and 10 μg/mL chloramphenicol (Solarbio; catalog no. C8050) in sterile water for 7 days before FMT.

For the microbial transplantation, 20 mg fresh stool from 4 pigs (5 mg/pig) was diluted 25-fold and homogenized in sterile prereduced 0.1 M potassium phosphate buffer (pH 7.2) containing 10% glycerol. The supernatant was then dispensed to cryotubes and immediately transferred to an environment at −80°C. The fecal suspension was administered orally by gavage to the recipient mice at a dosage of 0.2 mL/mouse once daily after the combined antibiotic treatment for 28 days.

To validate the function of HDCA in regulating host lipid metabolism, the mice were randomly assigned to the following 2 groups (*n* = 10): (i) the control group, which received an oral administration of oleic acid (Solarbio; catalog no. 08291) at a dosage of 0.2 mL/mouse once daily under HFD conditions; and (ii) the HDCA group, which received an oral administration of oleic acid at a dosage of 0.2 mL/mouse and 100 μM HDCA (Epsilon; catalog no. Z-028) once daily for 28 days.

### RNA preparation and quantitative real-time PCR.

The total RNA was extracted from tissues and cells using TRIzol reagent (Invitrogen, Life Technologies, Grand Island, NY, USA) according to the manufacturer’s instructions. The quality and purity of the total RNA were assessed using a microspectrophotometer (Nano100; Nano Drop Technologies, Wilmington, DE, USA) and an Agilent 2100 bioanalyzer (Agilent, CA, USA). The cDNA templates were synthesized from 1 μg of total RNA using a high-capacity cDNA reverse transcription kit (Thermo Fisher Scientific, Waltham, MA, USA). Detailed information on qPCR was previously described (27). The 18S rRNA gene was used as a housekeeping gene, and the primer sequences are listed in Table S1 in the supplemental material.

### Hematoxylin and eosin staining.

Paraffin-embedded sWAT sections (5 μm) were stained with hematoxylin and eosin (H&E) as previously described (22). The adipose tissues were fixed with 4% formaldehyde for 48 h. The fixed tissues were dehydrated in an ethanol gradient from 75% to 100% and embedded in paraffin. The embedded tissues were sectioned (5 μm), stained with H&E, and imaged using an Evos XL Core cell imaging system (Life Technologies).

### Cell culture and differentiation *in vitro*.

Mouse 3T3-L1 preadipocytes were obtained from Peking Union Medical College Hospital (Beijing, China) and incubated at 37°C in a humidified 5% CO_2_ atmosphere in Dulbecco’s modified Eagle medium (DMEM; Lonza, Switzerland) containing 10% fetal bovine serum (FBS) and 1% penicillin-streptomycin. Upon confluence (day 0), the medium was replaced with adipogenic differentiation medium (DM) containing 10% FBS, 1% penicillin-streptomycin, 5 μg/mL insulin, 0.4 μg/mL dexamethasone, 0.5 mM 3-isobutyl-1-methyl-xanthene (IBMX), and 1 μM rosiglitazone. The culture was incubated at 37°C in 5% CO_2_ for 4 days. The medium was then replaced with DMEM supplemented with 10% FBS and 5 μg/mL insulin for another 2 days. After differentiation, the cells were harvested for subsequent experiments.

To validate the function of HDCA in regulating lipid metabolism in 3T3-L1 cells, 3T3-L1 cells were treated with 5 μM, 15 μM, and 25 μM HDCA throughout the whole differentiation region, and the cells were harvested for subsequent experiments. To validate the function of HDCA in regulating lipolysis in 3T3-L1 cells, successfully differentiated 3T3-L1 cells were treated with 5 μM, 25 μM and 50 μM HDCA for 2 h, harvested for a Western blot analysis, and harvested for glycerin determination.

To validate the function of PI (Yuanye, Shanghai, China; catalog no. Y35772) and PC (Sigma; catalog no. P3556) in regulating cholesterol metabolism *in vivo*, we used HepG2 cells to mimic the process *in vitro*. HepG2 cells were incubated at 37°C in a humidified 5% CO_2_ atmosphere in DMEM (Lonza, Switzerland) containing 1% FBS and 1% penicillin-streptomycin. HepG2 cells were treated with 10 μM and 20 μM PC with fatty acid-free bovine serum albumin (Applygen, China) for 24 h after starvation for 12 h, and the cells were harvested for subsequent experiments. HepG2 cells were also treated with 0.1 μM, 0.5 μM, and 1 μM PI (Yuanye, China) with fatty acid-free bovine serum albumin for 24 h after starvation for 12 h, and the cells were harvested for subsequent experiments.

### Oil red O staining.

To determine the changes in the expression of candidate genes potentially required for lipogenesis and lipolysis after the HDCA treatment during 3T3-L1 preadipocyte differentiation into white adipocytes, the cells were seeded in a 12-well plate (1 × 10^4^ cells/well). Intracellular lipid accumulation was visualized by Oil Red O (Solarbio, Beijing, China) staining on day 8 (55). The cells were washed with Dulbecco phosphate-buffered saline (DPBS) and fixed with 4% formaldehyde for 15 min at room temperature. Then, the cells were washed with distilled water and 60% isopropanol and stained with Oil Red O solution (60% isopropanol in water) for 15 min at room temperature. After rinsing with distilled water and 60% isopropanol, the cells were photographed under a microscope (Leica, Wetzlar, Germany).

### Western blot analysis.

The adipose tissues and cells were lysed in T-PER tissue protein extraction reagent (Thermo Fisher Scientific, Waltham, MA, USA) and M-PER mammalian protein extraction reagent (Thermo Fisher Scientific, Waltham, MA, USA) supplemented with a protease inhibitor cocktail (Roche, Indianapolis, IN, USA), respectively. The total proteins (20 to 50 μg) were separated using 10% SDS-PAGE and transferred to polyvinylidene difluoride (PVDF) membranes (Millipore, Madison, WI, USA). After blocking in Tris-buffered saline with Tween 20 (TBST) with 5% skim milk for 2 h at room temperature, the membranes were incubated with the primary antibody at 4°C overnight. GAPDH (glyceraldehyde-3-phosphate dehydrogenase; 1:5,000; CST, Danvers, MA, USA) was used as the loading control. Horseradish peroxidase-conjugated goat anti-rabbit IgG (1:5,000) was used as a secondary antibody, and the immunoreactive bands were detected using a FluorChem M fluorescent imaging system (Tanon 5200; Tanon Science & Technology Co., Ltd., Shanghai, China) with Pierce enhanced chemiluminescence (ECL) Western blotting substrate (Thermo Scientific Pierce, Rockford, IL, USA). The primary antibodies used in the present study included p-AMPKα (1:2,000; Bioss, Beijing, China), AMPKα1 (1:2,000; CST, Beijing, China), p-SREBF2 (1:2,000; Affinity, USA), SREBF2 (1:2,000; Affinity, USA), HMGCR (1:2,000; Solarbio, China), and GAPDH (1:2,000; CST, Beijing, China).

### Lipid and bile acid determination.

Lipidomics profiling of serum and iWAT was performed according to a previously described protocol (56, 57). Lipids were extracted from animal tissues and serum using a modified version of Bligh and Dyer’s method (56). The lipid quantification procedure, internal standards, and bioinformatics analysis were previously described (27).

The bile acid profiles in the fecal samples and serum from pigs and mice were quantified using ultraperformance liquid chromatography coupled with triple quadrupole mass spectrometry (UPLC-TQMS; Waters, Milford, MA) according to a protocol as previously described (58).

### Measurement of the free glycerol concentration and total cholesterol concentration.

The supernatant glycerol concentrations in 3T3-L1 cell culture after the HDCA administration were measured by using a glycerol assay (Applygen, Beijing, China) according to previously described protocols (59). The free glycerol concentrations were divided by the intracellular total protein concentrations for normalization, and the fold change of these ratios is displayed in the respective graph. The total cholesterol concentration in HepG2 cells after the PC administration was measured by using a total cholesterol (TC) content assay kit (Boxbio, Beijing, China) according to standard protocols.

### Statistical analysis.

The statistical analyses were performed using Student’s *t* test for the screening of differentially expressed genes (DEGs) and qPCR analysis (2-sided, unpaired). Two-sided Welch’s *t* test and Benjamini-Hochberg false-discovery rate (FDR) correction were used in the two-group analysis. Analysis of variance (ANOVA) with the Tukey-Kramer test and Benjamini-Hochberg correction was chosen for the multiple-group analysis. All data are reported as the means ± standard errors of the means (SEMs). The statistical significance of the differences between the groups is denoted as *, *P *< 0.05; **, *P *< 0.01; and ***, *P *< 0.001.

### Data availability.

The 16S rRNA gene sequencing raw data are available at https://figshare.com/articles/dataset/16S_rDNA_sequencing_data_for_WT_and_UCP1_pigs_under_CD_and_HFHC_diet/20677401/1 and https://figshare.com/articles/dataset/16_rDNA_sequencing_data_for_WT_and_UCP1_pigs_under_Chow_diet_condition/21641369/1.
