# Supplementary material for: Fecal Microbiota Was Reshaped in UCP1 Knock-In Pigs via the Adipose-Liver-Gut Axis and Contributed to Less Fat Deposition
Source: Microbiol Spectr. 2023 Jan 23;11(1):e03540-22. doi: 10.1128/spectrum.03540-22 (PMC9927592; doi:10.1128/spectrum.03540-22)
Supplement: Supplemental file 1 — Supplemental material. Download spectrum.03540-22-s0001.pdf, PDF file, 4.1 MB [file spectrum.03540-22-s0001.pdf]

Supplemental figure legends:

Figure S1. LEfSe analysis of WT and UCP1 pigs at each growth point under CD conditions.

Figure S2. UCP1 expression in WAT from pigs prevents HFHC diet-induced obesity and fecal microbiota under HFHC conditions. (A) H&E pictures of sWAT from CD-fed WT pigs and HFHC diet-fed WT and UCP1 pigs after 13 months of HFHC diet consumption. (B) qPCR analysis of gene expression in sWAT from HFHC-fed WT and UCP1 pigs expressed as relative units using the $2^{-\Delta\Delta Ct}$ method; C. LEfSe analysis of CD-fed WT pigs, HFHC-fed WT pigs and UCP1 pigs after 3 months (C), 8 months (D) and 13 months (E) of HFHC diet treatment. *$P < 0.05$, **$P < 0.01$, ***$P < 0.001$.

Figure S3. FMT of fecal microbes from UCP1 pigs prevents diet-induced obesity. (A, B) The average food intake of UCP1 and WT group pigs under CD conditions (A) and HFD conditions (B). (C) Relative organ weights in the WT and UCP1 groups under CD conditions and HFD conditions.

Figure S4. Relative abundance of *Unclassified_Erysipelotrichaceae*, *Turicibacter*, *Roseburia*, *rc4-4*, *Dorea*, *[Ruminococcus]* and *[Eubacterium]* under CD and HFHC conditions.

Figure S5. HDCA administration reduces obesity. (A) Design of the animal study. (B) The average food intake in the HDCA and control groups. (C) Relative organ weights in the HDCA and control groups (n = 7). (D) Representative Oil O strain pictures of 3T3-L1 cells treated with 5 μM, 15 μM and 25 μM HDCA during whole white adipose differentiation. (E) The relative content of oil O in (D). (E, F) Representative Oil O staining pictures showing successful adipocyte differentiation after the HDCA treatment.

Figure S6. UCP1 expression in WAT changes lipids in sWAT, serum and fecal microbiota in pigs. (A) The relative expression of the *NPC1L1* gene in the gut; (B) Venn plot showing the changed lipid species in iWAT and serum between WT and UCP1 pigs from 6.5 months of age after acute cold exposure under CD conditions. (C) The relative expression of genes involved in cholesterol and bile acid metabolism in HepG2 cells treated with PI. *$P < 0.05$.

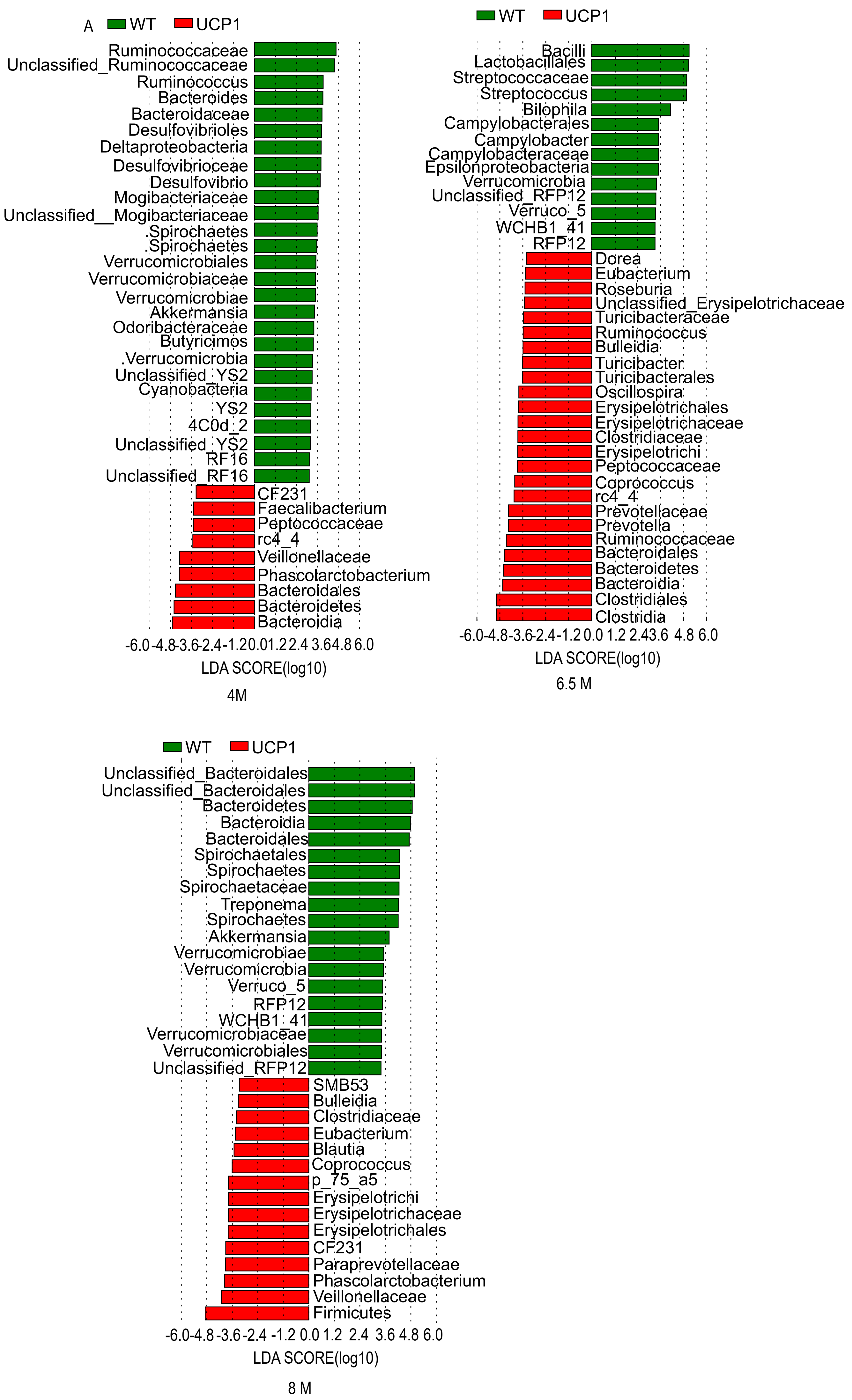
A
WT
UCP1
Ruminococcaceae
Unclassified_Ruminococcaceae
Ruminococcus
Bacteroides
Bacteroidaceae
Desulfovibrioles
Deltaproteobacteria
Desulfovibrioceae
Desulfovibrio
Mogibacteriaceae
Unclassified__Mogibacteriaceae
.Spirochaetes
.Spirochaetes
Verrucomicrobiales
Verrucomicrobiaceae
Verrucomicrobiae
Akkermansia
Odoribacteraceae
Butyricimos
.Verrucomicrobia
Unclassified_YS2
Cyanobacteria
YS2
4C0d_2
Unclassified_YS2
RF16
Unclassified_RF16
CF231
Faecalibacterium
Peptococcaceae
rc4_4
Veillonellaceae
Phascolarctobacterium
Bacteroidales
Bacteroidetes
Bacteroidia
-6.0 -4.8 -3.6 -2.4 -1.2 0.0 1.2 2.4 3.6 4.8 6.0
LDA SCORE(log10)
4M
WT
UCP1
Bacilli
Lactobacillales
Streptococcaceae
Streptococcus
Bilophila
Campylobacterales
Campylobacter
Campylobacteraceae
Epsilonproteobacteria
Verrucomicrobia
Unclassified_RFP12
Verruco_5
WCHB1_41
RFP12
Dorea
Eubacterium
Roseburia
Unclassified_Erysipelotrichaceae
Turicibacteraceae
Ruminococcus
Bulleidia
Turicibacter
Turicibacterales
Oscillospira
Erysipelotrichales
Erysipelotrichaceae
Clostridiaceae
Erysipelotrichi
Peptococcaceae
Coprococcus
rc4_4
Prevotellaceae
Prevotella
Ruminococcaceae
Bacteroidales
Bacteroidetes
Bacteroidia
Clostridiales
Clostridia
-6.0 -4.8 -3.6 -2.4 -1.2 0.0 1.2 2.4 3.6 4.8 6.0
LDA SCORE(log10)
6.5 M
WT
UCP1
Unclassified_Bacteroidales
Unclassified_Bacteroidales
Bacteroidetes
Bacteroidia
Bacteroidales
Spirochaetales
Spirochaetes
Spirochaetaceae
Treponema
Spirochaetes
Akkermansia
Verrucomicrobiae
Verrucomicrobia
Verruco_5
RFP12
WCHB1_41
Verrucomicrobiaceae
Verrucomicrobiales
Unclassified_RFP12
SMB53
Bulleidia
Clostridiaceae
Eubacterium
Blautia
Coprococcus
p_75_a5
Erysipelotrichi
Erysipelotrichaceae
Erysipelotrichales
CF231
Paraprevotellaceae
Phascolarctobacterium
Veillonellaceae
Firmicutes
-6.0 -4.8 -3.6 -2.4 -1.2 0.0 1.2 2.4 3.6 4.8 6.0
LDA SCORE(log10)
8 M

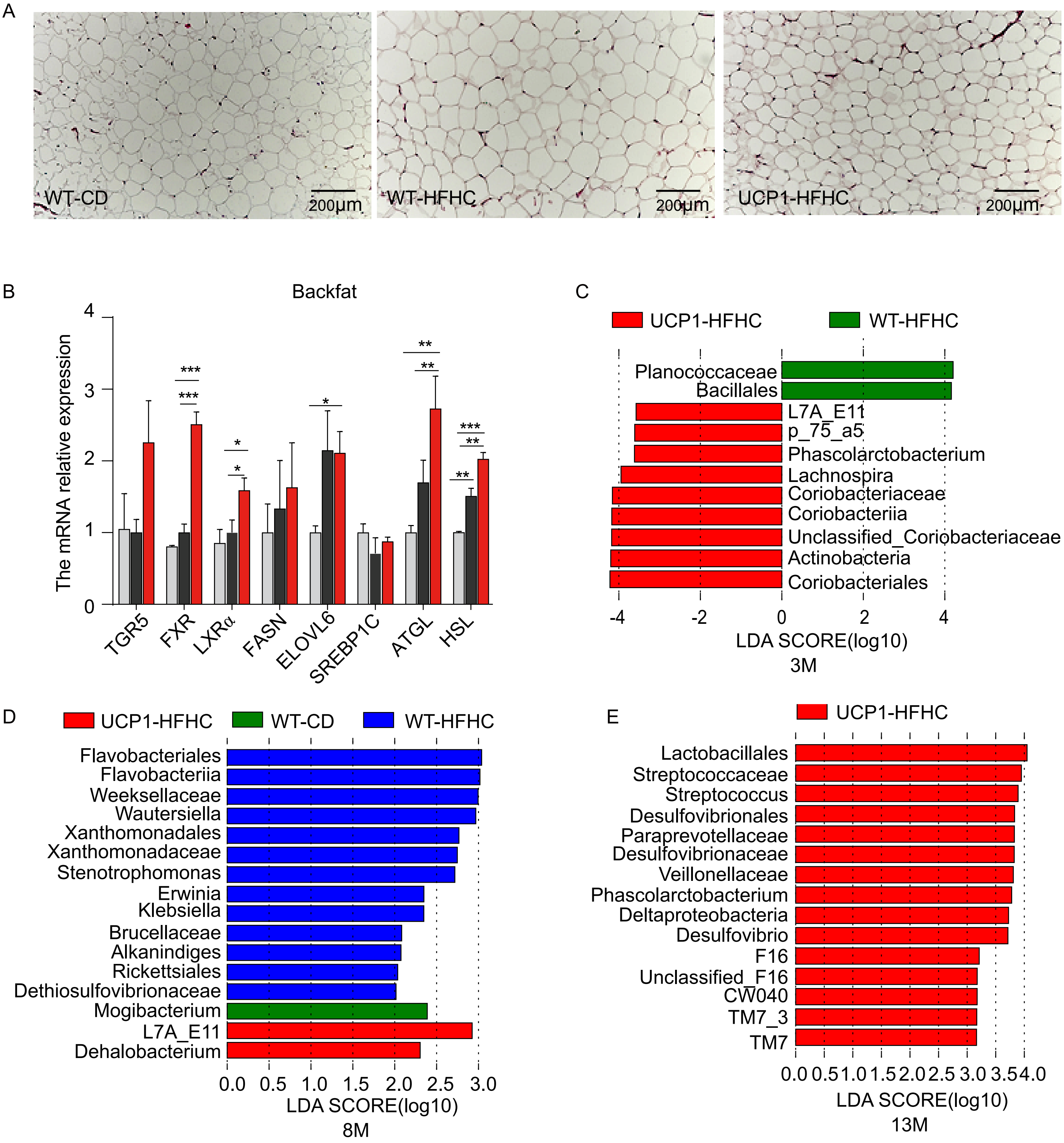

A
WT-CD
200μm
WT-HFHC
200μm
UCP1-HFHC
200μm
B
Backfat
The mRNA relative expression
TGR5
FXR
LXRα
FASN
ELOVL6
SREBP1C
ATGL
HSL
C
UCP1-HFHC
WT-HFHC
Planococcaceae
Bacillales
L7A_E11
p_75_a5
Phascolarctobacterium
Lachnospira
Coriobacteriaceae
Coriobacteriia
Unclassified_Coriobacteriaceae
Actinobacteria
Coriobacteriales
LDA SCORE(log10)
3M
D
UCP1-HFHC
WT-CD
WT-HFHC
Flavobacteriales
Flavobacteriia
Weeksellaceae
Wautersiella
Xanthomonadales
Xanthomonadaceae
Stenotrophomonas
Erwinia
Klebsiella
Brucellaceae
Alkanindiges
Rickettsiales
Dethiosulfovibrionaceae
Mogibacterium
L7A_E11
Dehalobacterium
LDA SCORE(log10)
8M
E
UCP1-HFHC
Lactobacillales
Streptococcaceae
Streptococcus
Desulfovibrionales
Paraprevotellaceae
Desulfovibrionaceae
Veillonellaceae
Phascolarctobacterium
Deltaproteobacteria
Desulfovibrio
F16
Unclassified_F16
CW040
TM7_3
TM7
LDA SCORE(log10)
13M

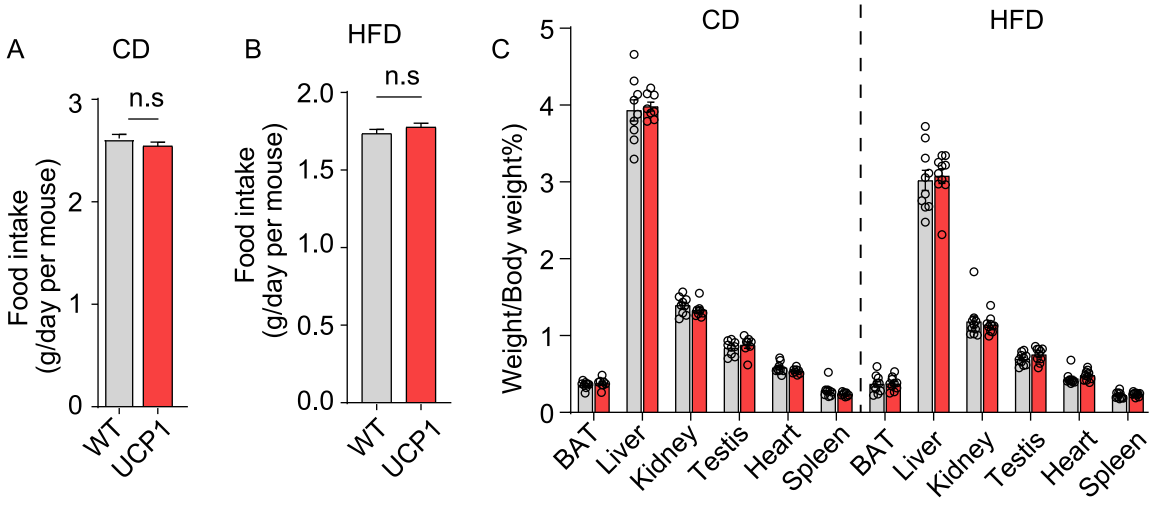

A
CD
n.s
Food intake (g/day per mouse)
0
1
2
3
WT
UCP1
B
HFD
n.s
Food intake (g/day per mouse)
0.0
0.5
1.0
1.5
2.0
WT
UCP1
C
CD
HFD
Weight/Body weight%)
0
1
2
3
4
5
BAT
Liver
Kidney
Testis
Heart
Spleen
BAT
Liver
Kidney
Testis
Heart
Spleen

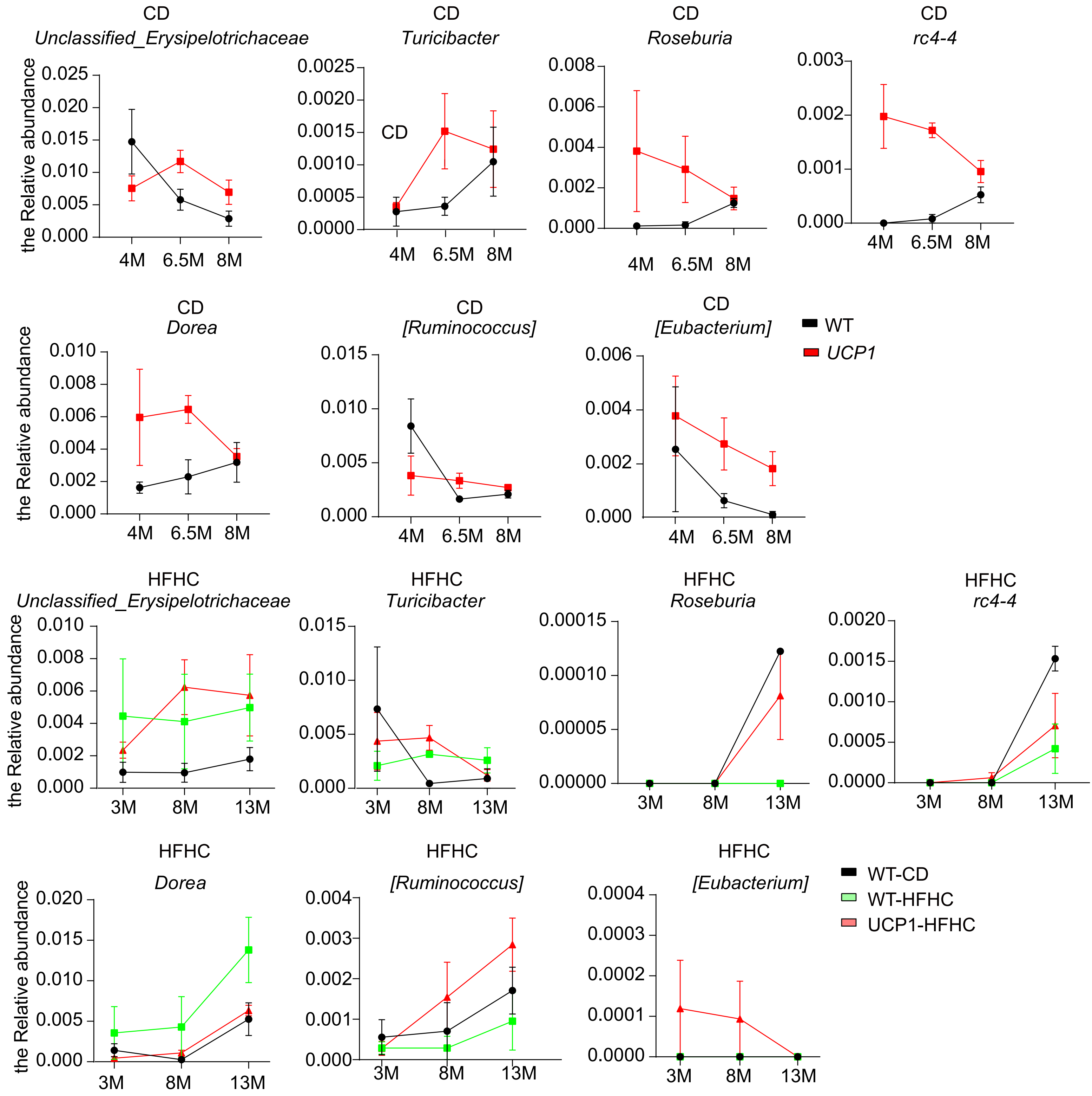
CD
Unclassified_Erysipelotrichaceae
CD
Turicibacter
CD
Roseburia
CD
rc4-4
CD
Dorea
CD
[Ruminococcus]
CD
[Eubacterium]
WT
UCP1
HFHC
Unclassified_Erysipelotrichaceae
HFHC
Turicibacter
HFHC
Roseburia
HFHC
rc4-4
HFHC
Dorea
HFHC
[Ruminococcus]
HFHC
[Eubacterium]
WT-CD
WT-HFHC
UCP1-HFHC
the Relative abundance
4M
6.5M
8M
3M
8M
13M

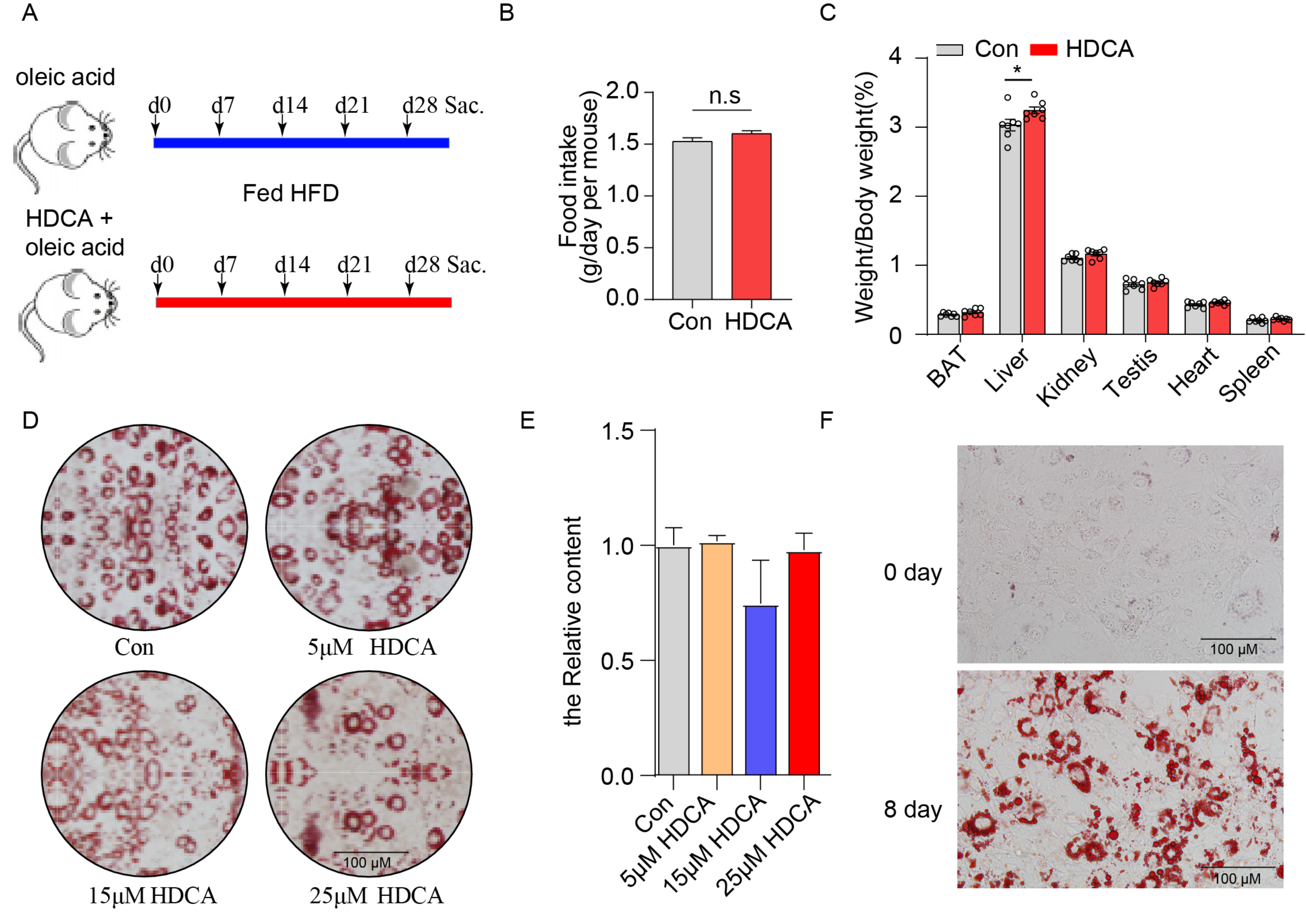
A
oleic acid
d0 d7 d14 d21 d28 Sac.
Fed HFD
HDCA +
oleic acid
d0 d7 d14 d21 d28 Sac.
B
Food intake
(g/day per mouse)
n.s
Con HDCA
C
Con HDCA
Weight/Body weight(%)
BAT Liver Kidney Testis Heart Spleen
D
Con
5μM HDCA
15μM HDCA
25μM HDCA
100 μM
E
the Relative content
Con
5μM HDCA
15μM HDCA
25μM HDCA
F
0 day
8 day
100 μM

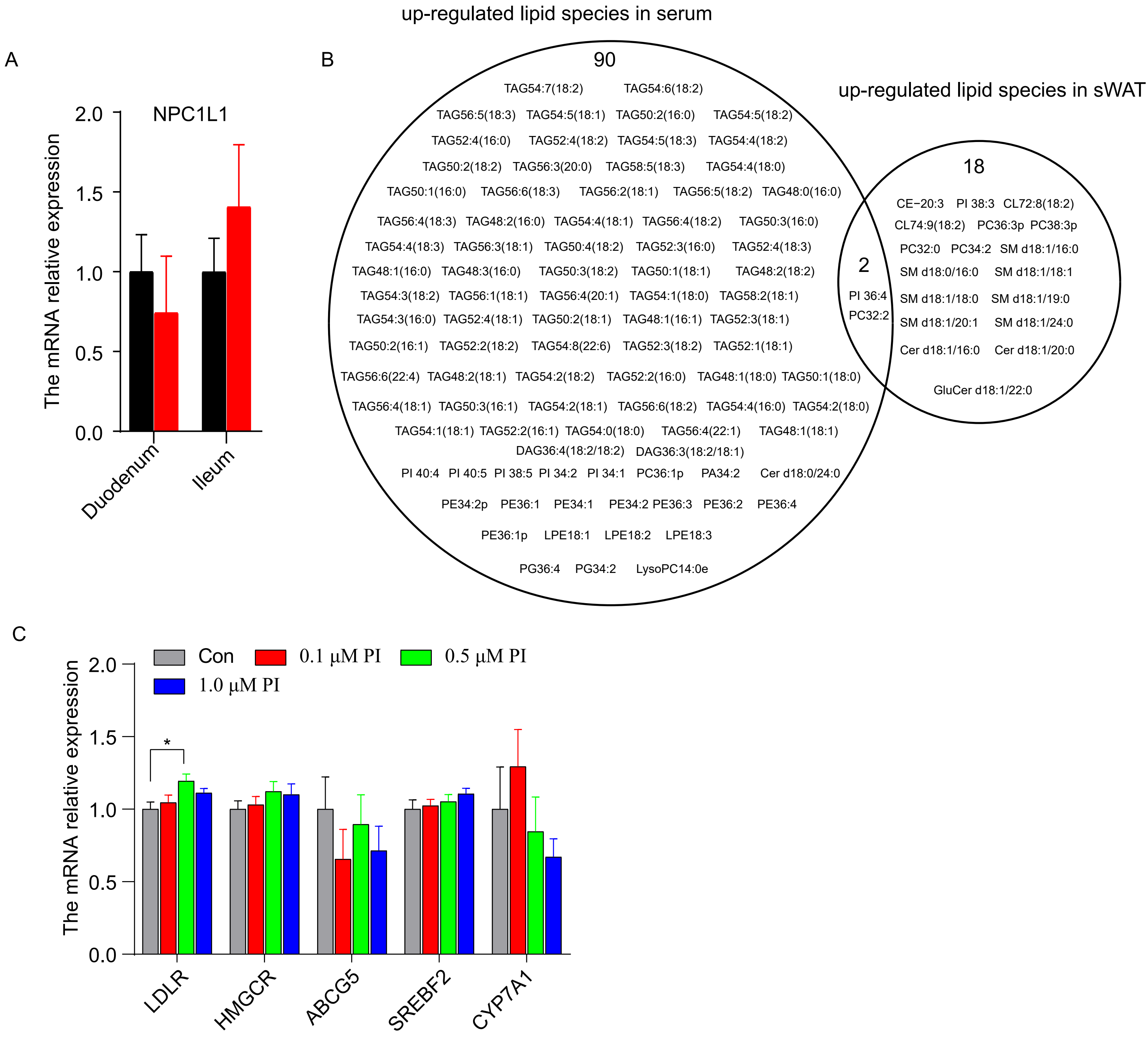
up-regulated lipid species in serum
A
B
NPC1L1
The mRNA relative expression
2.0
1.5
1.0
0.5
0.0
Duodenum
Ileum
90
TAG54:7(18:2) TAG54:6(18:2)
TAG56:5(18:3) TAG54:5(18:1) TAG50:2(16:0) TAG54:5(18:2)
TAG52:4(16:0) TAG52:4(18:2) TAG54:5(18:3) TAG54:4(18:2)
TAG50:2(18:2) TAG56:3(20:0) TAG58:5(18:3) TAG54:4(18:0)
TAG50:1(16:0) TAG56:6(18:3) TAG56:2(18:1) TAG56:5(18:2) TAG48:0(16:0)
TAG56:4(18:3) TAG48:2(16:0) TAG54:4(18:1) TAG56:4(18:2) TAG50:3(16:0)
TAG54:4(18:3) TAG56:3(18:1) TAG50:4(18:2) TAG52:3(16:0) TAG52:4(18:3)
TAG48:1(16:0) TAG48:3(16:0) TAG50:3(18:2) TAG50:1(18:1) TAG48:2(18:2)
TAG54:3(18:2) TAG56:1(18:1) TAG56:4(20:1) TAG54:1(18:0) TAG58:2(18:1)
TAG54:3(16:0) TAG52:4(18:1) TAG50:2(18:1) TAG48:1(16:1) TAG52:3(18:1)
TAG50:2(16:1) TAG52:2(18:2) TAG54:8(22:6) TAG52:3(18:2) TAG52:1(18:1)
TAG56:6(22:4) TAG48:2(18:1) TAG54:2(18:2) TAG52:2(16:0) TAG48:1(18:0) TAG50:1(18:0)
TAG56:4(18:1) TAG50:3(16:1) TAG54:2(18:1) TAG56:6(18:2) TAG54:4(16:0) TAG54:2(18:0)
TAG54:1(18:1) TAG52:2(16:1) TAG54:0(18:0) TAG56:4(22:1) TAG48:1(18:1)
DAG36:4(18:2/18:2) DAG36:3(18:2/18:1)
PI 40:4 PI 40:5 PI 38:5 PI 34:2 PI 34:1 PC36:1p PA34:2 Cer d18:0/24:0
PE34:2p PE36:1 PE34:1 PE34:2 PE36:3 PE36:2 PE36:4
PE36:1p LPE18:1 LPE18:2 LPE18:3
PG36:4 PG34:2 LysoPC14:0e
2
PI 36:4
PC32:2
up-regulated lipid species in sWAT
18
CE−20:3 PI 38:3 CL72:8(18:2)
CL74:9(18:2) PC36:3p PC38:3p
PC32:0 PC34:2 SM d18:1/16:0
SM d18:0/16:0 SM d18:1/18:1
SM d18:1/18:0 SM d18:1/19:0
SM d18:1/20:1 SM d18:1/24:0
Cer d18:1/16:0 Cer d18:1/20:0
GluCer d18:1/22:0
C
Con
0.1 μM PI
0.5 μM PI
1.0 μM PI
*
LDLR
HMGCR
ABCG5
SREBF2
CYP7A1

Supplementary Table 1 Primers sequences

Primers		Species	Sequence
TGR5	Forward primer	pig	CCAGACATCATCGCCAGAC
	reversed primer		GGTGCTGTTGGGTGTCATC
FXR	Forward primer		GGGATGAGCTTTGTGTCGTT
	reversed primer		TCCCATCTCTTTGCATTTCC
LXRA	Forward primer		GGCTTCCACTACAACGTGCT
	reversed primer		TCAGGCGGATCTGTTCTTCT
SREBP1C	Forward primer		CACACCCAGGTCCAAAGTG
	reversed primer		TGTTTTCATGGTCGTCAGGA
FASN	Forward primer		CTCATCACTGAGGCCACACA
	reversed primer		ACGGAGGAGAAGATCACGAAG
ELOVL6	Forward primer		AATGGATGCAGGAAAACTGG
	reversed primer		GGCCTTTGGTCATCAAAATG
ATGL	Forward primer		CGAACTCAAGAGCACCATCA
	reversed primer		AACCCTGCTTGCACATCTCT
HSL	Forward primer		GTCCTCGTCAGGCTCATCTC
	reversed primer		AGGTAAGGCTCGTGGGATTT
LDLR	Forward primer		TGGAAGAGAAATGTGGGAGAA
	reversed primer		CTCCAAGACTCAGGAATGCAG
HMGCR	Forward primer		GAAGCTCTGCCCTTTTTCCT
	reversed primer		AAGAGCATCGAGGGTGAATG
ABCG5	Forward primer		CACGTTGCAGATCGACTGAT
	reversed primer		TGGCAGTCATGCAGTCTAGG
SREBF2	Forward primer		GTGCAGCCTCAGATCATCAA
	reversed primer		CACCTGCTTAATGGGCACTT
CYP7A1	Forward primer		CAATTTGGTGCCAATCCTCT
	reversed primer		ATCACTGGGGTCAATGCTTC
CYP27A1	Forward primer		ACATGGACCTGTGGAAGGAG
	reversed primer		AGAATTGGTGGGCTATGTCG
TGR5	Forward primer	Human	CATGCACTTGGTCCACTTGT
	reversed primer		ATCTTGGTCCTGGGGACAG
FXR	Forward primer		GATGGGAATGTTGGCTGAAT
	reversed primer		CCTGCATGACTTTGTTGTCG
LXRA	Forward primer		TCAGAGAGGAAGCCAGGATG
	reversed primer		ATAGCTCGTTCCCCAGCAT

LDLR	Forward primer		TGTCTGTCACCTGCAAATCC
	reversed primer		AACTGCCGAGAGATGCACTT
HMGCR	Forward primer		TCCCTGGGAAGTCATAGTGG
	reversed primer		AGGATGGCTATGCATCGTG
ABCG5	Forward primer		GGCAGATCATGTGCATCCTA
	reversed primer		GCAGGACGTAGGAGAAGCAG
SREBF2	Forward primer		AATGATCACGCCAACATTCA
	reversed primer		TACTGTCTGCACCTGCTGCT
TGR5	Forward primer	mouse	CACTTGACCCCCAACTTTTG
	reversed primer		GCAAAGAACAGGGAGCTGAC
FXR	Forward primer		CCAACAGACCCTCCTGGAT
	reversed primer		AAGAAACATGGCCTCCACTG
LXRA	Forward primer		GGATAGGGTTGGAGTCAGCA
	reversed primer		GCTCAGCACGTTGTAATGGA
SREBP1C	Forward primer		GGAGCCATGGATTGCACATT
	reversed primer		GCTTCCAGAGAGGAGGCCAG
ACACA	Forward primer		GAGAGGGGTCAAGTCCTTCC
	reversed primer		ACATCCACTTCCACACACGA
ATGL	Forward primer		GACGGTGGCATCTCAGACAA
	reversed primer		GCACATCTCTCGAAGCACCA
HSL	Forward primer		GTCAGGTGTCTTTGCGGGTA
	reversed primer		TGGCTTGTGCGGAAGAAGAT
18s	Forward primer	pig/human/mouse	GTAACCCGTTGAACCCCATT
	reversed primer		CCATCCAATCGGTAGTAGCG
